# Comparison of WP-2 and MOCNESS plankton samplers for measuring zooplankton biomass in the Barents Sea ecosystem

**DOI:** 10.1093/plankt/fbae065

**Published:** 2024-11-28

**Authors:** Hein Rune Skjoldal, Johanna Myrseth Aarflot, Tor Knutsen, Peter H Wiebe

**Affiliations:** Institute of Marine Research, Ecosystem Processes Research Group, PO Box 1870 Nordnes, N-5817 Bergen, Norway; Institute of Marine Research, Ecosystem Processes Research Group, PO Box 1870 Nordnes, N-5817 Bergen, Norway; Institute of Marine Research, Plankton Research Group, PO Box 1870 Nordnes, N-5817 Bergen, Norway; Woods Hole Oceanographic Institution, Woods Hole, MA 02543-1050, USA

**Keywords:** gear intercomparison, size fractions, zooplankton biomass, zooplankton nets, zooplankton sampling

## Abstract

Zooplankton in the Barents Sea has been monitored on an annual autumn survey since the late 1980s, using vertical WP-2 and oblique Multiple Opening and Closing Net and Environmental Sensing System (MOCNESS) tows over the water column. Sampling with MOCNESS is used to describe the vertical distribution and more frequent sampling with WP-2 (~3:1) to describe the horizontal distribution. We use here a large cumulative data set of 874 MOCNESS and 2850 WP-2 stations with data on size-fractioned dry-weight biomass to compare the two zooplankton sampling gears. MOCNESS is consistently collecting more biomass of the large size fraction (>2 mm screen size) by ~20% and less of the small fraction (<1 mm) by ~30% compared to WP-2. This is interpreted to reflect more extrusion of small plankton and less avoidance by larger plankton with the MOCNESS. The data set has been collected by three research vessels. There was a difference in vertical speed in oblique tows of MOCNESS among the ships but no clear effect on volume filtered per unit time. This demonstrates operational consistency and suggests the use of a constant flow factor (distance per flowmeter count) when calculating results over the time series. The issue of calibration of traditional flowmeters on oblique tows needs further examination.

## INTRODUCTION

The Institute of Marine Research (IMR) in Norway has been studying zooplankton in the Barents Sea since 1979 (Skjoldal, 2023). From 1985 onward, a standard procedure was used in which each net sample is split into two halves, one used for determination of dry weight in three size fractions, while the other half is preserved with formalin and stored for later species enumeration (Melle *et al*., 2004; Hassel *et al*., 2020). Since 1986, zooplankton sampling has been routinely included in monitoring surveys in the autumn (combined 0-group fish and acoustic capelin surveys, broadened in 2004 into an ecosystem survey; Eriksen and Prozorkevich, 2011; Michalsen *et al*., 2013; Eriksen *et al*., 2018). The autumn surveys are carried out jointly with the Polar Branch of the Russian Federal Research Institute of Marine Fisheries and Oceanography (PINRO) in Murmansk, Russia (Jakobsen and Ozhigin, 2011). Results from the zooplankton monitoring have been used to describe changes in the Barents Sea ecosystem in relation to climate and trophic interactions with pelagic fish stocks, notably capelin *Mallotus villosus* (Skjoldal *et al*., 1992, 2022; Dalpadado *et al*., 2002, 2003, 2012, 2014, 2020; Stige *et al*., 2014). In recent years, the joint results from the IMR and PINRO have been used in integrated ecosystem assessments of the Barents Sea by an International Council for the Exploration of the Sea (ICES) working group (WGIBAR, 2021).

The IMR has used a sampling design combining two zooplankton sampling gears, the vertical WP-2 net (Fraser, 1968; Skjoldal *et al*., 2019) and the Multiple Opening and Closing Net and Environmental Sensing System (MOCNESS) multiple net sampling system (Wiebe *et al*., 1985; Skjoldal *et al*., 2000, 2018). The rationale for this has been partly practical. MOCNESS is somewhat more sensitive to bad weather conditions when operated from the IMR research vessels, and processing of the samples is more laborious due to the large number of nets with samples that are processed separately. The WP-2 net is simpler to operate with vertical tows from near-bottom to surface. With more frequent sampling of stations with WP-2, better horizontal resolution of the distribution of zooplankton biomass is obtained. Vertical resolution (commonly sampling 5–7 depth intervals over the water column) and more representative samples of rarer and larger organisms, such as krill and amphipods, are obtained with oblique MOCNESS tows taken less frequently (e.g. Dalpadado and Skjoldal, 1996; Eriksen *et al*., 2016).

The zooplankton of the Barents Sea can be broadly separated into boreal species found in the Atlantic water of the southern part and Arctic species associated with Arctic water in the northern part (Tande, 1991; Eiane and Tande, 2009; Dalpadado *et al*., 2012). The copepods *Calanus finmarchicus* and *C. glacialis* are two (largely) herbivorous species that dominate the mesozooplankton biomass in these two domains, respectively (Aarflot *et al*., 2018). The *Calanus* species combined are estimated to make up ~80% of the total recorded zooplankton biomass in the Barents Sea (Aarflot *et al*., 2018; Skjoldal and Aarflot, 2023). In autumn samples, the biomass-dominant older copepodite stages (predominantly C5) are contained in the medium size fraction (Skjoldal, 2021). Small copepod species such as *Oithona* spp. and *Pseudocalanus* spp. are contained in the small size fraction, while the large fraction contains large chaetognaths, amphipods and krill (Skjoldal, 2021).

The IMR procedure with splitting of samples and biomass determination in size fractions was used as the basis for the comparison of a range of different zooplankton sampling gear in a sea-going workshop reported by Skjoldal *et al*. (2013). MOCNESS and WP-2 net were included in this intercomparison study. They were found to give comparable results (as were also other nets such as the MultiNet). However, the number of replicates was limited, and the study did not allow small differences in sampling performance to be resolved. Gjøsæter *et al*. (2000) reported a comparison of WP-2 and MOCNESS with data from the Barents Sea. They found that WP-2 net collected relatively more of the smallest size fraction, and MOCNESS collected more of the largest size fraction. These differences countered each other, and there was no significant difference in total zooplankton biomass obtained with the two gears.

Here, we expand on the comparison of Gjøsæter *et al*. (2000). They used data from the autumn surveys in the Barents Sea from 1988 to 1997. Their study included a total number of 115 stations where both WP-2 and MOCNESS were used to collect zooplankton biomass. We have included data after 1997 (up to 2015), which gives us a considerably larger data set to do the comparison. The objective of this paper is to quantify differences in the sampling performance of the two gears. This is important to allow better evaluation of results on zooplankton biomass, abundance and composition across studies and geographical regions. With ongoing climate change affecting zooplankton communities (e.g. Richardson, 2008), this aspect of methodology is especially important for evaluating changes over time with data from different sources and zooplankton sampling gears.

## MATERIALS AND METHODS

### Sampling

#### Research vessels

The sampling with WP-2 and MOCNESS on autumn cruises has been done largely with three IMR research vessels: RV “Johan Hjort” (1989–2015) and two versions of RV “G.O. Sars,” the old ship (1989–2002) and the new ship (2003–2015) (Table I). WP-2 and MOCNESS were also used on RV “Eldjarn” in 1988–1990. Other ships (RV “Michael Sars” and RV “Jan Mayen”/“Helmer Hansen”) have also taken part in the autumn cruises in the Barents Sea, but they have used only WP-2 and not MOCNESS.

**Table I:** Number of sampling stations with zooplankton samples collected by three IMR research vessels on autumn cruises in the Barents Sea between 1989 and 2015: RV “Johan Hjort,” 1989–2015; RV “G.O. Sars” (GOS), old vessel, 1989–2002; and RV “G.O. Sars,” new vessel, 2003–2015

Year	WP-2				MOCNESS				
	Johan Hjort	GOS Old	GOS New	Total	Johan Hjort	GOS Old	GOS New	Total	Same stations
1989		24		73		14		14	11
1990		15		71		13		27	8
1991	37	39		76	17	15		32	25
Sum 89–91	**37**	**78**		**220**	**17**	**42**		**73**	**44**
1992	64	49		113	10	15		25	21
1993	56	69		125	15	22		37	9
1994	75	87		162	28	23		51	26
1995	40	68		108	18	24		42	10
1996	33	55		88	25	29		54	
1997	66	49		115	30	12		42	11
1998	54	116		170	17	27		44	27
1999	69	91		160	16	24		40	29
2000	55	77		132	18	37		55	23
2001	44	93		137	22	30		52	7
2002	56	48		104	19	27		46	7
2003	75		12	87	31		4	35	5
2004	148			148	35			35	4
2005	31		97	128	15		34	49	23
2006	68		78	146	19		22	41	40
2007	52		88	140	14		21	35	31
2008	22		74	96	10		10	20	10
2010	85		58	143	13		13	26	24
2011	102			102	16			16	15
2012			69	69			10	10	10
2013	81		80	161	18		11	29	22
2014	80		17	97	40		6	46	3
2015	100		19	119	28		16	44	
Sum 92–15	**1456**	**802**	**592**	**2 850**	**457**	**270**	**147**	**874**	**357**

The sums in 1989 and 1990 include additional stations with zooplankton sampling by RV “Eldjarn.” Two sampling gears have been used: WP-2 net (0.25 m^2^) towed vertically, and MOCNESS (1 m^2^) towed obliquely (or stepwise in 1992–1994) over the water column from near the seafloor to the surface. MOCNESS was operated with 333 μm nets in the three first years shown separately (1989–1991). Both systems were used with 180 μm mesh in 1992–2015, and this constitutes the main comparison of the two nets with the “all stations” data set (*n* = 2 850 for WP-2 and *n* = 874 for MOCNESS). The last column gives the number of stations where both WP-2 and MOCNESS were used for comparison with the “same stations” data set (*n* = 357). Sums for the first three (1989-1991) and subsequent years (1992-2015) are shown with bold font.

#### WP-2 net

The WP-2 net (Fraser, 1968; Wiebe and Benfield, 2003) has been a 56 cm diameter version (0.25 m^2^, length of net 261 cm) equipped with 180 μm mesh net during the whole sampling period (Skjoldal *et al*., 2019; Hassel *et al*., 2020). The net has been hauled vertically with a speed of ~0.5 m s^−1^. The WP-2 has been used without a flowmeter, and the results are calculated from vertical towing distance assuming 100% filtration efficiency. Two tows were taken at each station, from near bottom to surface and from 100 m depth to surface. Here, we are using the data for the whole water column for comparison with vertical profiles obtained with MOCNESS. “Near-bottom” is typically within 10 m of the recorded echo-sounder depth at the station. The mean distance between the lower sampling depth and bottom depth was ~9 m (SD 2 m) over all sampling stations, with small differences between the three ships (Table II). The sampling gap represented ~3.5% of the water column (Table II).

**Table II:** Sampling “gap” as distance between lower sampling depth and bottom depth as mean values with standard deviation (SD) for annual values for stations with zooplankton sampling with MOCNESS and WP-2 for each of the three research vessels (name and ID number) and combined

	MOCNESS			WP-2		
Ship	Mean	SD	%	Mean	SD	%
Johan Hjort (12)	23.4	7.3	8.8	7.8	2.1	3.2
Old G.O. Sars (15)	25.4	8.7	10.1	9.2	1.6	3.8
New G.O. Sars (10)	25.1	6.5	8.7	10.6	0.6	3.8
All ships	24.6	6.4	9.2	8.6	1.6	3.5

% is the sampling gap as percentage of the mean water depth.

#### MOCNESS

MOCNESS has been the 1 m^2^ version (Wiebe *et al*., 1976, 1985), towed obliquely from near-bottom to the surface for most of the time (from 1995 onward) (Wiebe *et al*., 2015). It was used with 333 μm nets in 1989–1991 and with 180 μm mesh from 1992 onward. “Near-bottom” for the MOCNESS tows means usually within 20–30 m from the recorded depth at the station. How close to the bottom the MOCNESS is operated has varied around a mean of ~25 m (SD ~7 m), again with small differences among the three vessels (Table II).

MOCNESS is equipped with a modified TSK flowmeter (Tsurumi-Seiki-Kosakusho Co., Ltd) and sensors that measure tilt angle of the net frame when it is towed (Longhurst *et al*., 1966; Wiebe *et al*., 1985). The MOCNESS is designed to give a square opening of 1 m × 1 m (1 m^2^) when the frame is at an angle of 45^o^ to the vertical (Wiebe *et al*., 1976, 1985). There is also a pressure sensor that is used to record depth and a sensor that records when nets are successively opened and closed (one net is closed as the next is opened). The data from the sensors are sent to a deck unit via cable, and a computer program calculates the volume filtered for each net in depth intervals given by pressures and times when nets are opened and closed.

The flowmeter is calibrated and a factor converting flowmeter counts to distance towed is used to calculate volume filtered by a net, also using the information on the tilt angle of the net frame, the tow angle of the oblique tow and the vertical speed of the net (Wiebe *et al*., 1985). A flow factor (in units of meters per count) of 4.5 (value given in the MOCNESS manual; Anonymous, 2003) was used consistently on all (three) research vessels (see below) between 1989 and 2007 or 2008 (Table S1, in Supplementary material). MOCNESS was not used on cruises in 2009 (Table I). From 2010, a flow factor of 4 was used on both Johan Hjort and G.O. Sars (new), with a change to a higher value of ~6 for G.O. Sars from 2013 (6.15 in 2013, 5.9 in 2014 and 6.0 in 2015; Table S1). Various versions of software have been used since the 1980s. Much effort has been spent by IMR technicians, scientists and instrument personnel ensuring that the MOCNESS units have functioned properly, and technical details on the operation of MOCNESS are generally kept in logbooks from the cruises. The information includes stored data on depth, flowmeter counts, tilt angle, horizontal and vertical speed and calculated filtration volumes for each of the nets of a MOCNESS profile. More information on the operation of the MOCNESS on cruises in the Barents Sea, and on the calculation of volume filtered by nets, is provided in a supplementary document (Supplementary material, part B).

#### MOCNESS performance

The data we report here (see section Ship effects in Results) suggest there could be a “ship effect” affecting the MOCNESS results. Flowmeter calibration and operational procedures on the research vessels may have contributed to the effect. Calibration of the flowmeters has been done on occasion by towing the MOCNESS along a one nautical mile distance in both directions to eliminate or reduce the effect of water currents (Table S6, Supplementary part B).

We have examined all logbooks from the cruises for information on the operation of the MOCNESS sampler. The raw data with recorded information on flowmeter counts, pressure, frame angle and time exist for most of the MOCNESS tows. We reran computations of the volume filtered by nets from some of the raw data to check the information stored in the IMR database. We did this for cruises where the volume filtered per tow time (see subsequent text; Fig. S1B in supplementary material) was either low or high compared to the median. We also checked data for cruises where the ratio MOCNESS/WP-2 for zooplankton biomass (based on cruise average values) was deviating on the low or high side (see Fig. 9). In most cases where volume was recalculated, the existing data on volume filtered were confirmed to be correct within a few percent deviation. However, we detected some errors for single sampling stations that were removed from the data set.

There was an issue with double flowmeter counts for some of the results. This is where a magnetic response switch records twice (each half-rotation) and not once per rotation. The problem of double counts was noted in the logbook from the cruise with RV G.O. Sars (old) in 2001, and the results from that cruise had been corrected by dividing the calculated volume filtered from flow counts by factor 2 (to correct from use of flow factor 4.5 to 2.25 m per count). The results from RV Johan Hjort in 2007 showed volume filtered per tow-time about twice as high as other results (Fig. S1), while the ratio of MOCNESS/WP-2 for the cruise average values of zooplankton biomass was ~0.5. Our assessment concluded that this was due to double counts (Table S8 in Supplementary part B), and the MOCNESS results for biomass for this cruise have been multiplied by 2 (volume too high by factor 2 gives biomass per volume too low by factor 2).

Estimated volume filtered and tow time were used to indicate tow performance for individual MOCNESS tows. These two variables were calculated as sums for all the nets used in a vertical MOCNESS profile except the upper net (usually from 25 m to surface; due to inconsistent recording of closing time for this net). We use the ratio of volume filtered per unit time (m^3^ min^−1^) as a further index of tow performance (Fig. S1). Volume filtered and tow time were high in the first 3 years (1992–1994) but shifted down (by about a factor of 2) from 1995 onward. This corresponded to a shift from stepwise to oblique tows (Wiebe *et al*., 2015). For the samples from 1995 with oblique tows, the volume filtered varied around a mean of 720 m^3^ (SD 348, median 659 m^3^), while the tow time varied around a mean of 15 min (SD 6.8) (Table S2).

The average water depth at the MOCNESS sampling stations was 266 m (SD 88), while the average sampling depth was 242 m (SD 86; Table S2). The frequency distributions of the depth variables were close to normal, indicated by low values of skewness and kurtosis (<0.3; Table S2). The performance variables for the MOCNESS tows were also close to a normal distribution, with some skewness to high values for filtered volume (Table S2). Water depth and sampling depth were strongly correlated and explained ~50% of the variance of sampling volume and tow time (*R*^2^ = 0.48–0.53; Table S3, Fig. S2).

**Fig. 1:**
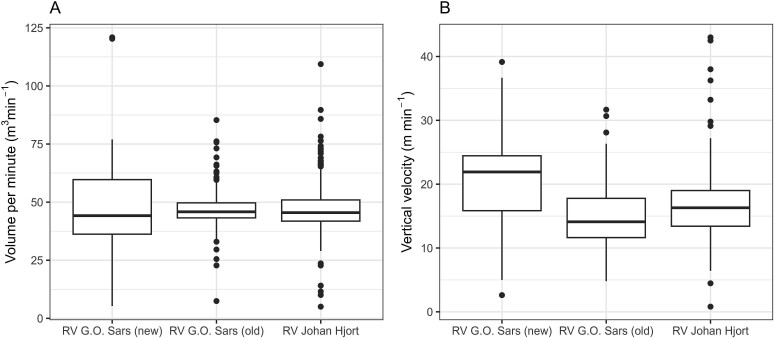
Volume filtered per time (**A**) and vertical velocity (**B**) for MOCNESS profiles (minus upper net) obtained with oblique tows from the three research vessels, G.O. Sars (new) (2003–2015), G.O. Sars (old) (1995–2002) and Johan Hjort (1995–2015). Box-whisker plots show median (horizontal bar), 25–75 percentiles (box), 5–95 percentiles (vertical line) and statistical outliers (dots).

The volume filtered per unit time varied around a mean of 47 m^3^ min^−1^ (SD 7.6) (Table S2) and showed little difference among the three research vessels (Fig. 1A). Some cases where volume filtered per time (m^3^ min^−1^) was either exceptionally low or high (see Fig. S1B) appeared to be due to an error in the time recorded in the database. Thus, the volume recorded appeared not to deviate when compared to the other ship (the same year) or across years. Removing the lowest and highest values of volume per minute (4.5% of stations in each end, keeping values between 30 and 70 m^3^ min^−1^), shifted the frequency distribution of towing time from deviating from, to being close to, a normal distribution, as indicated by skewness and kurtosis values (from a high kurtosis value of 5.8 to <1 for both kurtosis and skewness for the combined data for all three ships; Table S2). This reduced data set (90% of the stations) included the 5–95 percentiles for Johan Hjort and the old G.O. Sars, while removing a somewhat larger fraction of the samples for the new G.O. Sars, which displayed more variation (Fig. 1A). With the reduced data set, there was no significant difference in volume filtered per time between the three vessels (Table S4).

The mean vertical hauling speed was 15 m min^−1^ (SD 4.5; Table S2). There was a significant difference between the three ships, with the speed being higher by 36% on average for the new G.O. Sars compared to Johan Hjort (19.8 versus 14.6 m min^−1^) and lower by 13% for the old G.O. Sars (12.7 m min^−1^) (Fig. 1B, Fig. S9, Table S5, Table S7). The higher vertical speed for the new G.O. Sars (meaning that the MOCNESS was brought up more quickly) was associated with lower volume filtered for an oblique tow compared to the other ships (Figs S2 and S3).

The results on volume filtered were recalculated from stored raw data for the MOCNESS oblique tows taken during the cruises with Johan Hjort and G.O. Sars (new) in 2007 and 2008. The output included the mean values of the recorded frame angle and the calculated tow angle (Fig. S8) for each net of a vertical profile. The results of the reanalysis are given in Supplementary part B.

The frame angle was related to towing speed with strict positive relationships (*R*^2^ = 0.59–0.89 for the two vessels; Fig. S11). This is as expected: with increasing speed, the frame is towed with a flatter orientation (more horizontally) in the water. The mean values of the frame angle (θ) were 44–45 and 48–52 degrees for G.O. Sars and Johan Hjort, respectively. Reflecting the higher vertical speed of the MOCNESS on the new G.O. Sars (Fig. 1B), the tow angle (φ) was significantly higher for this ship than for Johan Hjort (15.2–15.4 versus 10.6–12.3; Table S8). There was an inverse relationship between the two angles (Fig. S10), suggesting that higher vertical speed caused the frame to tilt less relative to the vertical.

**Fig. 2:**
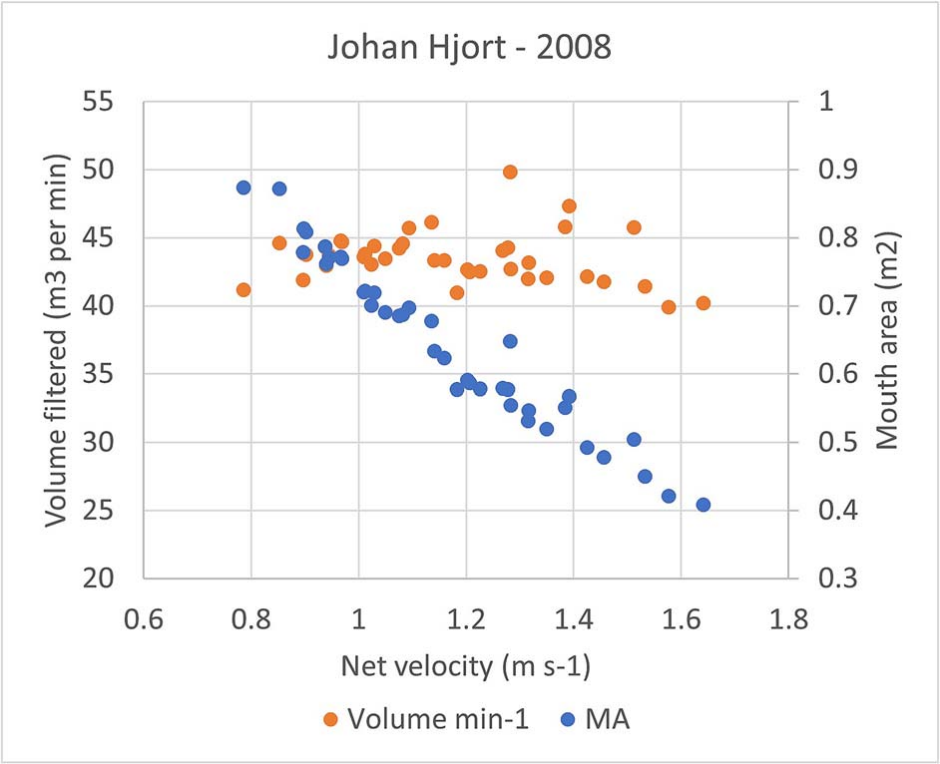
Mouth area (MA) and volume filtered per unit time versus net speed for MOCNESS nets from 11 stations (vertical profiles) taken during cruise with RV Johan Hjort in 2008.

The higher frame angle at higher towing speed caused the effective mouth area of the net to decrease [as a function of cos (θ + φ)]. The volume filtered per time is the product of speed times mouth area. The decrease in mouth area with increased towing speed was of similar magnitude as the increase in speed (a factor of ~2 decrease in area for a factor 2 increase in speed). This counters the effect of speed to give a stable volume filtered per unit time relatively unaffected by changes in towing speed (Fig. 2, Fig. S12).

The data for G.O. Sars in 2008 were reprocessed with two alternative flow factors—4.5 and 6.0 m per flowmeter count. The choice of flow factor influences the calculated towing speed directly through multiplication (flow counts × flow factor). The two calculations of volume filtered gave a strictly linear relationship (Fig. S15) but with a slope that was ~10% higher than the proportionality factor for the ratio of the two flow factors [1.47 versus 1.33 (6.0/4.5)]. This extra effect was due to a difference in the calculated tow angle φ (Fig. S13). With higher towing velocity (tv, in the oblique direction) and a constant vertical velocity (vv, calculated from measured pressure independent of flow factor), the angle φ becomes smaller since it is calculated from the sine function: vv = tv * sin (φ) (Fig. S14). We have used factor 1.47 when recalculating results with flow factor 6.0 for cruises where the original data were calculated with flow factor 4.5 (Table S1): volume filtered multiplied by 1.47 and biomass per volume and per square meter divided by 1.47.

### Determination of biomass

After hosing the net with seawater to get zooplankton down into the cod end, the IMR procedure (Hassel *et al*., 2020; see Melle *et al*., 2004; Skjoldal *et al*., 2013; Skjoldal, 2023) is to empty the contents of the cod end into a tray to pick out gelatinous zooplankton [which are usually identified, counted and measured (length and/or volume) separately]. The sample is then split into two halves with a Motoda splitter (Motoda, 1959), where one-half is preserved with formalin and stored, while the other is used to determine size-fractioned dry weight biomass. The samples obtained with MOCNESS are larger than those with WP-2 due to larger sampling volume [by factor 10–15 from larger mouth opening (factor ~4) and oblique haul (factor ~3)]. For practical purposes (e.g. size of weighing trays), the biomass fraction of large MOCNESS samples is sometimes divided one or two more times (one-fourth or one-eighth fraction of the total sample) before processing for weighing.

The biomass sample (zooplankton suspended in seawater) is successively sieved through screens with 2000, 1000 and 180 μm mesh plankton gauze. The zooplankton retained on each screen is rinsed quickly with freshwater (to remove adherent saltwater) and transferred to pre-weighed aluminum trays. The samples are dried usually for 24 h at 65°C (to constant weight), stored frozen and then weighed with microbalance back at the IMR laboratory. The three zooplankton biomass fractions are denoted large (>2 mm), medium (1–2 mm) and small (<1 mm). The size here refers to the screens used in fractionation, but there is a general strict relationship with the size of the zooplankton organisms being screened (Skjoldal, 2021; Skjoldal and Aarflot, 2023).

The dry weight biomass of each MOCNESS sample (multiplied by 2, 4 or 8 dependent on the fraction of the total sample used for biomass determination) is divided by volume filtered by the net to get biomass in grams per cubic meter. Depth-integrated biomass m^−2^ is obtained by multiplying biomass m^−3^ with the depth interval sampled by a net and then summing the values for the various nets over the whole (sampled part) of the water column. For WP-2, depth-integrated biomass m^−2^ is obtained as the biomass collected in the vertically hauled net multiplied by 8 (factor 2 for fraction used for biomass determination and factor 4 to go from 0.25 m^2^ net opening to 1 m^2^).

### Data sets

For the comparisons of the sampling performance of MOCNESS and WP-2, we are using the data on depth-integrated biomass for the three size fractions and their sum, which is the total sampled biomass. At each sampling station, a single vertical tow with the WP-2 and/or a single oblique tow with MOCNESS were used. There is therefore a direct correspondence between the number of sampling stations and the number of individual tows for each of the two gears.

A total of nearly 1000 (981) MOCNESS tows have been collected on autumn cruises from 1989 to 2015, while the corresponding number of WP-2 samples is ~3000 (3072). Including cruises where MOCNESS was not used, the total number of WP-2 samples is nearly 4000 (3931) for the period 1989–2015. In this study, we used data from autumn cruises where both gears were used (Table I). MOCNESS was used with 333 μm mesh nets in the first years up to and including 1991 (the sums of the number of sampling stations in 1989–1991 are shown separately in Table I). The number of MOCNESS stations for 1992–2015 has been reduced (from 908 to 874) by removing 26 stations with a sampling gap of 10 m or more between the closure of the upper net and the surface, 3 stations where data on one or more fractions were missing and 5 stations where we assessed data to be wrong [G.O. Sars (old), 1994, stations #961, 964 and 967; Johan Hjort, 2000, station #693; Johan Hjort, 2006, station #834]. On average on autumn cruises each year since 1992, 38 MOCNESS tows were taken (varying from 10 to 55), while the average for WP-2 was 124 samples (varying from 69 to 170). No MOCNESS samples were taken in 2009 for technical reasons. For the WP-2 data set, two samples were removed due to missing data for fractions (2850 sampling stations for the years 1992–2015; Table I).

**Fig. 3:**
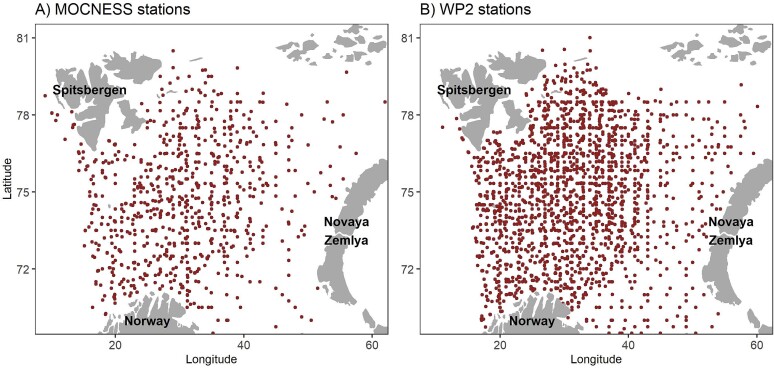
Map of sampling stations on autumn cruises in the Barents Sea in 1992–2015 where samples were collected with (A) MOCNESS and (B) WP-2 net. This is the “all stations” data set with 854 MOCNESS stations and 2850 WP-2 stations.

The autumn cruises are typically carried out with three research vessels from IMR and one to two vessels from the Russian institute PINRO (Michalsen *et al*., 2013; Eriksen *et al*., 2018). For practical reasons (ship time available, ice conditions, etc.) and shifting research priorities (e.g. due to changes in fish stocks), the cruise design has varied over time. However, in most cases, a regular sampling design has been followed, with similar spacing between cruise lines and sampling stations. The zooplankton sampling stations are also more or less evenly distributed, although a fixed station grid has not been used (Skjoldal *et al*., 2000). The design has been flexible, with about one MOCNESS station taken for every three WP-2 stations. This has ensured a wide spread of both MOCNESS and WP-2 stations (Fig. 3), where the fewer MOCNESS stations are dispersed regularly among the denser coverage of WP-2 stations. This justifies a comparison of results obtained with MOCNESS and WP-2 for all the stations across all the years. We denote this large data set “all stations.” After the quality evaluation and removing stations with missing data (e.g. one of the biomass fractions or the upper net with MOCNESS), the total numbers of samples for this data set are 874 MOCNESS and 2850 WP-2 (Table I).

MOCNESS and WP-2 have been used in parallel at a total of 357 sampling stations in the period 1992–2014 (no samples at the same station in 2015) when both gears were used with 180 μm mesh nets (Table I). We call this the “same stations” data set. The number of stations in this set varies from a low of 3 stations in 2014 to a high of 40 in 2006. Note that the two data sets are not independent since the “same stations” are also included in the “all stations” data set.

### Statistical analyses

Preliminary and exploratory data analyses were performed with Microsoft Excel. This included univariate descriptive statistics, correlations (Pearson *r*) and preparation of some of the figures. Box-whisker plots, scatter plots and linear regressions were done using R version 4.3.1 (R Core Team, 2023). Regressions between biomass obtained with MOCNESS and WP-2 were calculated as major axis (geometric mean) regressions, using log10-transformed biomass.

Statistical significance for the difference between mean biomass (or other variables) was tested using the *t*-statistic. This included testing the significance of Pearson correlation coefficients.

## RESULTS

### Comparisons across the “same stations” and “all stations” data sets

**Fig. 4:**
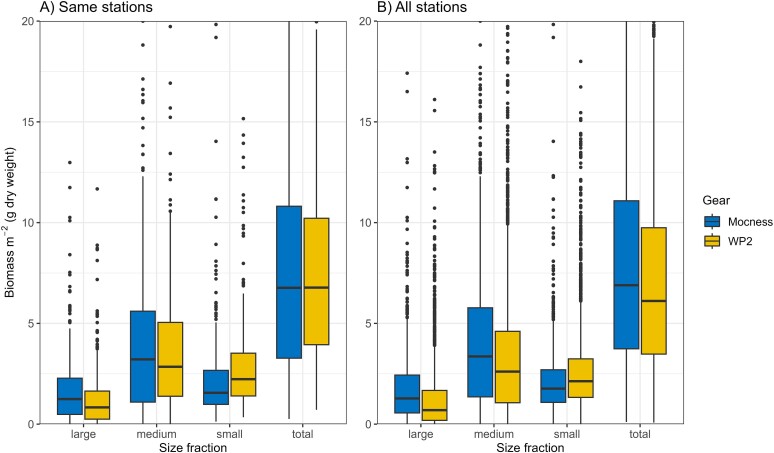
Zooplankton biomass (g dry weight m^−2^) in three size fractions and total for the “same stations” (A) and “all stations” (B) data sets of samples collected with MOCNESS and WP-2 net in the Barents Sea (1992–2015). Box-whisker plots show median (horizontal line), 25–75% quantiles (box), 5–95% quantiles (vertical line), and statistical outliers (circle symbol; note there is a cut-off at 20 g dry weight m^−2^ on the *y*-axis; see Fig. 6 for the full statistical distribution in the high end for total biomass of the “all stations” data set). Summary statistics are given in Table III.

In the comparison of depth-integrated biomass for the data set obtained at the same stations (1992–2014, *n* = 357 stations), MOCNESS collected more biomass of the large size fraction, while WP-2 collected more biomass of the small size fraction. MOCNESS collected more biomass also of the medium size fraction but to less extent (Table III, Fig. 4A). These differences were statistically significant (two-tailed *t*-test; *P* < 0.001 for the large and small fractions, *P* < 0.01 for the medium fraction). The higher and lower catches canceled each other to some degree, and the two gears gave quite similar estimates of the total biomass in this comparison (8.2 (MOCNESS) and 7.9 (WP-2) g dry weight m^−2^; Table III; not significantly different at 5% level).

**Table III:** Zooplankton dry mass (g m^−2^) for three size fractions—large > 2 mm; medium 1–2 mm; small < 1 mm—and total

	MOCNESS				WP-2				Difference MOCNESS-WP-2			
	Large	Medium	Small	Total	Large	Medium	Small	Total	Large	Medium	Small	Total
“Same stations” data set, *n* = 357												
Mean	1.71	4.31	2.21	8.23	1.26	3.7	2.91	7.87	0.45	0.61	-0.69	0.37
SD	1.82	4.46	2.23	6.82	1.54	3.71	2.56	6.03	1.61	3.74	2.6	6.32
CV	1.06	1.03	1.01	0.83	1.22	1	0.88	0.77				
Median	1.25	3.21	1.56	6.76	0.83	2.85	2.23	6.78	0.25	0.13	−0.49	−0.03
Minimum	0	0.03	0.12	0.26	0	0	0.34	0.71	−10.4	−14.1	−18.9	−33.7
Maximum	13.0	36.0	19.8	44.6	11.7	42.4	24.6	59.9	9.3	19.1	18.4	31.7
Kurtosis	9.2	9.6	23.1	4.7	11.2	35.3	18.6	21.1	11.2	5.6	17.6	6.3
Skewness	2.5	2.4	3.9	1.8	2.8	4.3	3.4	3.3	−0.33	1.19	−0.66	0.39
%	20.8	52.4	26.9	100	16	47.1	37	100				
												
“All stations” data set, *n* = 874 MOCNESS, *n* = 2 850 WP-2												
Mean	1.86	4.36	2.21	8.43	1.23	3.46	2.69	7.37	0.64	0.9	−0.48	1.06
SD	2.06	4.33	1.9	6.75	1.61	3.73	2.4	5.93				
CV	1.1	0.99	0.86	0.8	1.32	1.08	0.89	0.8				
Median	1.28	3.36	1.76	6.89	0.7	2.61	2.13	6.11	0.58	0.75	−0.37	0.78
Minimum	0	0	0.01	0.11	0	0	0	0.08				
Maximum	20.2	36.0	19.8	54.7	16.1	63.1	41.1	101				
Kurtosis	16.9	10.5	20.6	8	14.5	44.8	44.1	34.6				
Skewness	3.2	2.5	3.5	2.1	3.1	4.5	4.7	3.7				
%	22.1	51.7	26.2	100	16.6	46.9	36.5	100				

Summary statistics for two sample series: “same stations” and “all stations”. SD, standard deviation; CV, coefficient of variation (=SD/mean); %, percentage proportion for each size fraction to total biomass (=100%). The differences in biomass between MOCNESS and WP-2 are for the individual pairwise comparisons with the “same stations” data set and for the mean biomass values for the “all stations” data set.

**Fig. 5:**
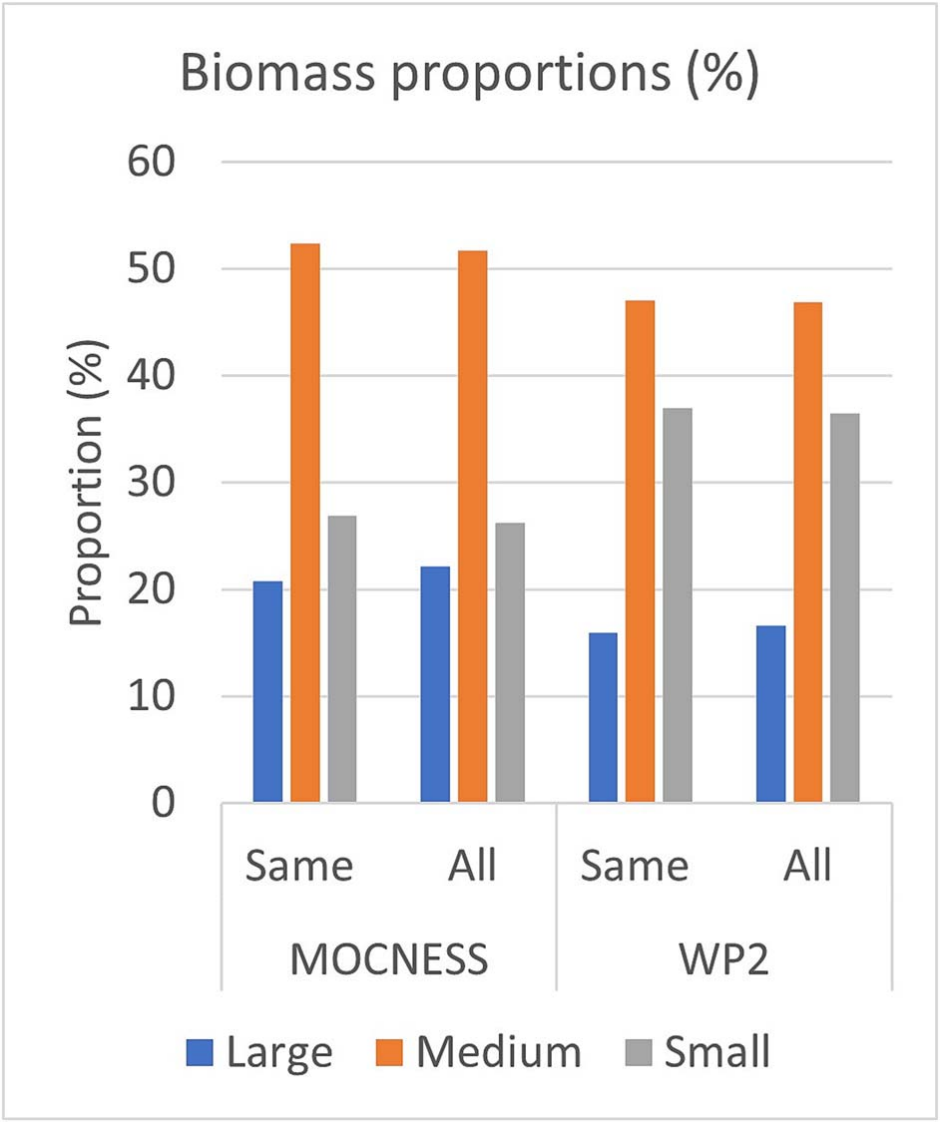
Proportion (%) of zooplankton biomass (as g dry weight m^−2^) in three size fractions collected with MOCNESS and WP-2 for the “same stations” and “all stations” data sets (1992–2015).

For the total data set from all stations (1992–2015), the comparison gave similar results (Table III). MOCNESS collected more zooplankton of the large and medium size fractions, while WP-2 collected more of the small fractions (Fig. 4B). In this larger data set, MOCNESS collected more total biomass than WP-2 (8.4 vs 7.4 g dw m^−2^). All differences were statistically significant (two-tailed *t*-test; *P* < 0.001). The relative distribution of biomass among the three size fractions was similar in the two data sets but consistently different between the two sampling gears (Figs 4 and 5). Using the larger data set (“all stations”), MOCNESS collected ~22% of the total biomass in the large fraction, 52% in the medium fraction and 26% in the small fraction. The corresponding numbers for the WP-2 were ~17% in the large, 47% in the medium and 37% in the small fraction. The relative differences for each fraction separately were that MOCNESS collected 51% more biomass of the large fraction, 26% more of the medium fraction and 18% less of the small fraction compared to WP-2. MOCNESS collected 14% more total zooplankton biomass than WP-2 (percentages based on mean values in Table III).

### Variability

**Fig. 6:**
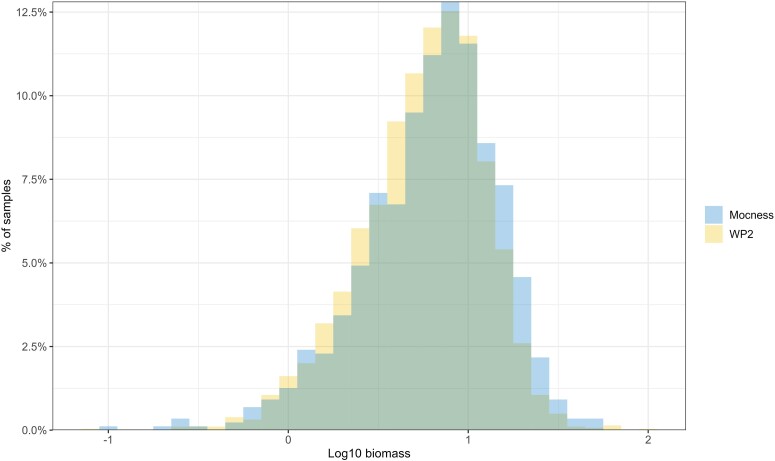
Frequency distributions of total zooplankton biomass (log10) collected with MOCNESS and WP-2 for the “all stations” data set (*n* = 874 and 2850 for MOCNESS and WP-2, respectively). The figure shows the large zone of overlap of the two sets of columns.

The variability of the data sets of depth-integrated zooplankton biomass was characterized by the standard deviation (SD) being of the same magnitude as the mean; the coefficient of variation (CV = SD/mean) was largest for the biomass of the large size fraction (1.06–1.32) and smallest for the small fraction (0.86–1.01; Table III). The CV was generally lower for the total biomass (0.77–0.83) than for the size fractions. The frequency distribution of biomass data was skewed with a tail of high values for both MOCNESS and WP-2 (Fig. 4). The asymmetry is reflected in positive skewness values of 1.8–4.7 for the three fractions as well as total biomass (Table III). Deviation from a normal distribution was also reflected in values of excess kurtosis (between 5 and 45; Table III). When log-transformed, the frequency distributions (for the “all stations” data set) were close to log-normal but with some distortion (overrepresentation) in the low end (Fig. 6; moderate negative skewness values between −0.5 and −1.3). The frequency distributions for MOCNESS and WP-2 were remarkably similar with the same general shape but with the MOCNESS distribution shifted to higher values compared to WP-2 (statistically significant higher log-normal mean, *P* < 0.01, *t*-test).

Some of the high biomass values for samples collected with MOCNESS and WP-2 might be interpreted as outliers in a statistical sense, although they are probably real features reflecting patchiness in plankton distribution. The high values come from a continuous statistical distribution (Fig. 6), which suggests that they are real. The maximum values recorded in the data series tended to be higher for WP-2 than for MOCNESS, with a maximum of 101 g dw m^−2^ for WP-2 and 55 g dw m^−2^ for MOCNESS (Table III). The skewness of the data (relative to a normal distribution) is reflected in median values being smaller than the means. The difference (median/mean, as percentage) was generally largest for the large size fraction (27–43%), lower for the medium and small size fractions (20–30%) and lowest for total biomass (14–18%; Table III).

**Fig. 7:**
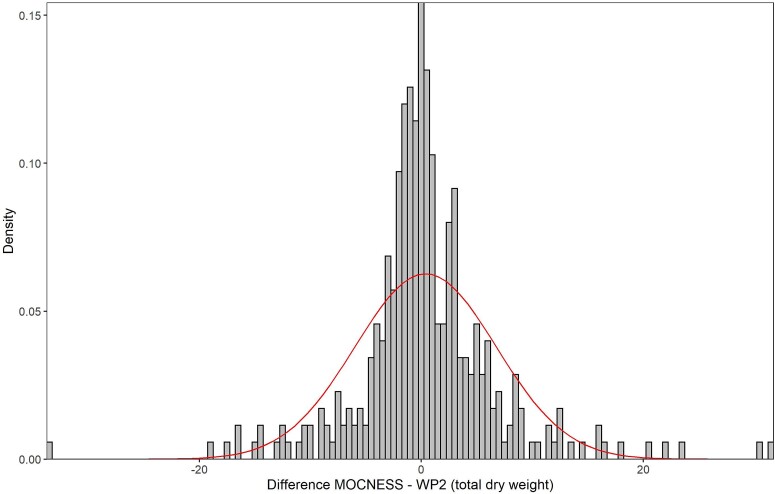
Frequency histogram for the difference in total zooplankton biomass (g dry mass m^−2^) in pairwise comparisons of MOCNESS and WP-2 for the “same stations” data set (*n* = 357). The curve is a normal distribution fitted to the data.

For the pairwise comparison of biomass collected with MOCNESS and WP-2 for the “same stations” data set, the SD for the difference (MOCNESS minus WP-2) was of the same magnitude as the SDs for each of the data series (MOCNESS and WP-2 separately) (Table III; note that the mean difference equals the difference between the two means). This reflects a wide scatter in the pairwise data set. The frequency distribution for the difference of total zooplankton biomass was symmetrical on a linear scale (skewness 0.4) but differed from a normal distribution in showing high kurtosis (6.3; indicating a sharper peak with heavy tails or outliers), with a median value of 0.0 g dw m^−2^ and spanning a range from −34 to +32 g dw m^−2^ (Fig. 7, Table III).

### Regressions

**Fig. 8:**
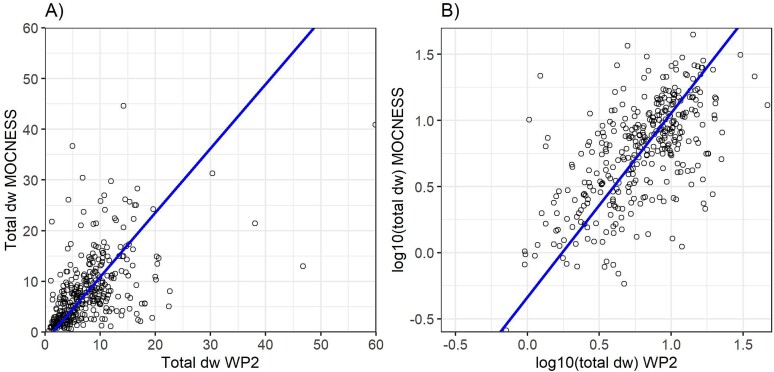
Plot of total zooplankton biomass (g dry weight m^−2^) collected with MOCNESS (*y*-axis) versus WP-2 (*x*-axis) at the same stations (*n* = 357). The same data are shown on linear (A) and logarithmic (B) scales. The lines are linear regressions (major axis; equations are given in Table IV).

Using the pairwise (“same stations”) data set, the MOCNESS and WP-2 biomass values regressed positively and significantly for each fraction and total biomass, although “only” 17% (small fraction) to 36% (medium fraction) of the variance were explained by the linear regressions (Table IV, Fig. 8). The regression slope was higher than one for the large and medium fractions (~1.35), showing higher catches for MOCNESS than WP-2, and was lower than one for the small fraction (0.87), showing lower catches for MOCNESS (Table IV). This is in general agreement with the comparisons of the sample means above. The scatter plots (shown for the total in Fig. 8A) show the statistical property of the data described above (Fig. 4), with a dense concentration of points at the lower end of the scale and a more dispersed scatter of points at the high end. A log transformation gives a more balanced presentation of the data points but with only a moderate increase in the variance explained by linear regression (from 27% to 38%; Fig. 8B).

**Table IV:** Results from linear regression analysis (major axis or geometric mean) for biomass of zooplankton collected with MOCNESS and WP-2 nets in pairwise comparisons for samples taken at the same stations

Fraction	*u*	*v*	*R* ^2^
Large	0.01	1.35	0.30
Medium	−0.72	1.37	0.36
Small	0.11	0.87	0.17
Total	−1.73	1.27	0.27
Total log10	−0.34	1.39	0.38

Biomass (MOC) = *u* + *v* * Biomass (WP-2), where *u* is intercept and *v* is slope. *R*^2^ is fraction of variance explained. *N* = 357.

**Fig. 9:**
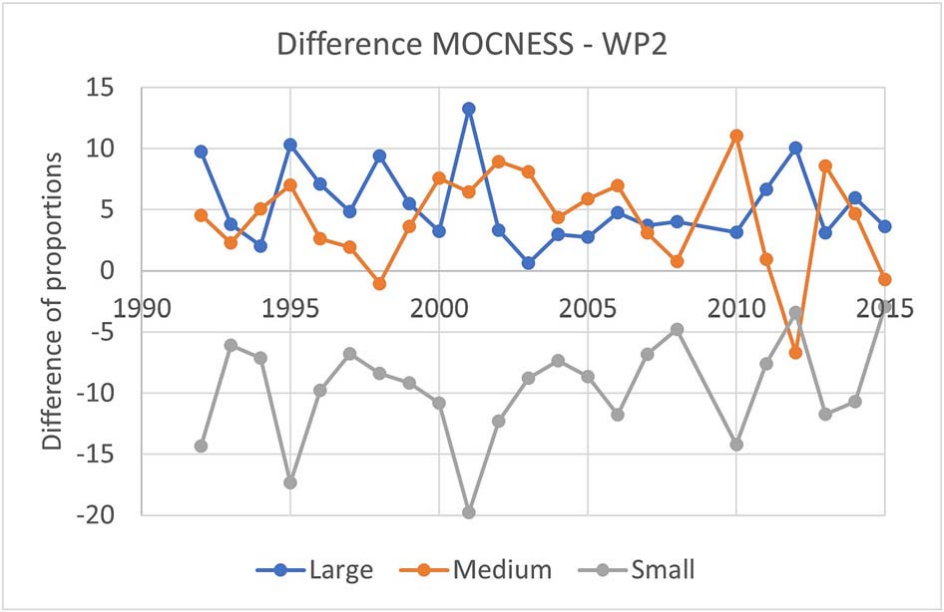
Difference in proportions (%) of biomass collected in three size fractions between MOCNESS and WP-2. Data are based on annual means (across RVs) for the “all stations” data set (see Table I). Note that data for 2009 are missing.

### Temporal and spatial consistency

Annual mean autumn biomass values (expressed as a percentage of the total) for samples collected with MOCNESS and WP-2 showed similar temporal patterns for each of the three size fractions over the time series from 1992 to 2015 (Fig. S4). Fluctuations and trends in the fractions were reflected rather consistently by the two sampling gears, as evidenced by significant positive correlations (*P* < 0.001) over the time series (*R*^2^ values of 0.72, 0.71 and 0.66 for the large, medium and small fractions, respectively). MOCNESS was always collecting more biomass of the large fraction and less biomass of the small fraction compared to WP-2 (Fig. 9). MOCNESS collected also more of the medium fraction than WP-2 in most cases (18 out of 23 years, with 4 years tied (within +/− one percent unit) and one year (2012) with a negative difference).

There was also spatial consistency when the “all stations” data set was broken down into four quadrants of the Barents Sea (SW, SE, NW and NE, split by 74^o^N and 30°E; Fig. S5). On a relative scale (percentage) and compared to WP-2, MOCNESS collected 33–57% more biomass in the large fraction, 15–35% more biomass in the medium fraction and 13–23% less biomass in the small fraction, as averages for the four regions. MOCNESS gave higher biomass for the total zooplankton, by 6–21% for the four regions.

### Ship effects

**Fig. 10:**
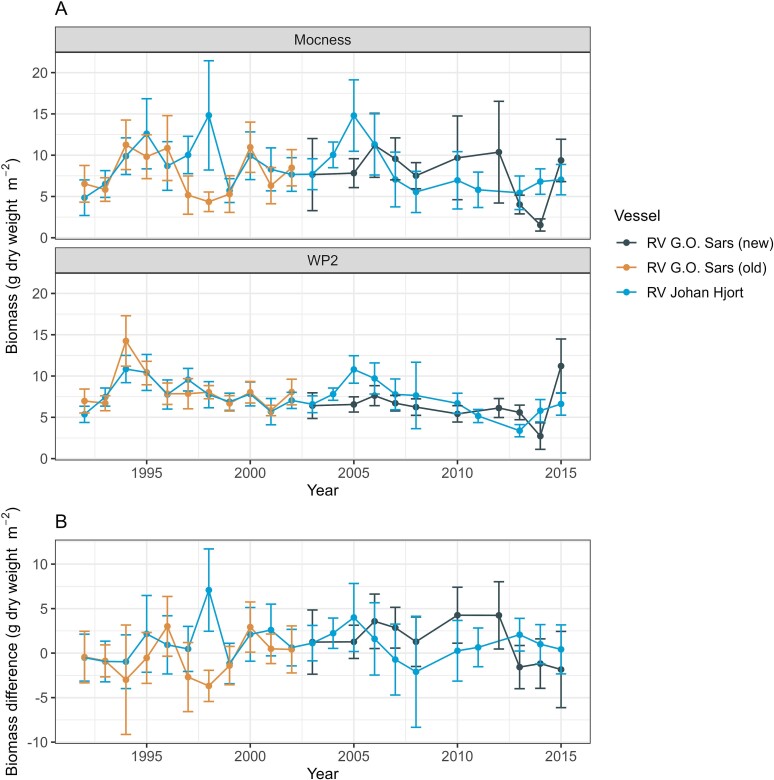
Annual autumn mean total zooplankton biomass obtained with WP-2 and MOCNESS (A), and difference between the annual means of MOCNESS minus WP-2 (B) for three research vessels, 1992–2015. The annual mean values by ship are based on the “all stations” data set; see Table I for number of stations. Vertical error bars are +/−2 standard error (SE), approximately equivalent to confidence intervals for single comparisons.

Preliminary data exploration suggested that there could be differences in results for MOCNESS and WP-2 obtained by the research vessels used to collect the data. Annual mean total biomass values obtained with WP-2 and MOCNESS, as well as the difference between them (MOCNESS minus WP-2), are shown in Fig. 10 for the three vessels separately. The WP-2 results give a “smoother” appearance than the MOCNESS results that show stronger fluctuations between years (Fig. 10A). This is reflected in the similarity in the pattern of variations in the MOCNESS results to that shown by the difference between MOCNESS and WP-2 (Fig. 10A and B; *r* (Pearson) = 0.67 between the series of MOCNESS data and the series of difference (*P* < 0.001), versus *r* = −0.16 for WP-2 data and the difference). The larger variation in MOCNESS is partly due to the lower number of sampling stations, with means of 15–25 stations annually for each of the three ships, compared to mean station numbers of 59–73 for WP-2 (see Table I). The variability is illustrated with box-whisker plots in Fig. S6. The confidence interval (CI, 95%) for each of the “ship and year” data sets of total zooplankton biomass was on average 36% (relative to mean biomass; range 16–79%) for MOCNESS, compared to a mean of 20% (range 9–62%) for WP-2. The corresponding data for the medium fraction gave mean CI values of 46% and 28% for MOCNESS and WP-2, respectively.

The larger (random) sampling error for MOCNESS (due to high sampling variance; see Discussion) complicates the detection of sampling bias in the MOCNESS-WP-2 comparisons. CIs (95%) for the annual mean MOCNESS and WP-2 values by ships are generally overlapping, and the CIs for the difference between them are also (with few exceptions) including the value zero (Fig. 10). MOCNESS collected more total biomass than the WP-2 net in most cases (27 out of 43 ship–year combinations) and more biomass also of the medium fraction (31 out of 43 cases). The difference between MOCNESS and WP-2 by year and ship for total biomass showed a distribution close to normal, indicated by low values of skewness and kurtosis (0.4 for both). The median difference was 0.62 g dw m^−2^ across the annual values by ships, with a mean of 0.73 g dw m^−2^ (SD 2.2), representing 9% compared to WP-2 total biomass. The medium size fraction showed similar temporal patterns to those of total biomass for the different ships (Fig. S7). The medium fraction made up ~50% of total biomass (Table V) and has been shown to correlate strongly with total biomass (*R*^2^ ~0.8; Skjoldal and Sperfeld, 2024). The difference MOCNESS minus WP-2 for the medium fraction was also close to normally distributed (skewness and kurtosis < 0.2), with mean and median values of 0.69 and 0.68 g dw m^−2^, representing 19% higher biomass in MOCNESS samples compared to WP-2.

**Table V:** Results for zooplankton biomass collected with MOCNESS by RVs Johan Hjort, old G.O. Sars and new G.O. Sars in two periods, 1992-2002 and 2003-2015.

		Proportion as % of total			Biomass ratio MOCNESS/WP-2			
Period	Ship	Large	Medium	Small	Large	Medium	Small	Total
1992–2002	Johan Hjort	25.5	49.4	25.1	1.50	1.23	0.83	1.14
	GO Sars Old	24.0	46.5	29.4	1.30	1.03	0.68	0.94
2003–2015	Johan Hjort	19.1	57.7	23.2	1.53	1.23	0.81	1.14
	GO Sars New	17.6	54.5	27.9	1.46	1.29	1.00	1.22

Results are given for mean proportions of biomass for three size fractions as % of total biomass collected with MOCNESS and as ratio of mean biomass collected with MOCNESS to mean biomass collected with WP-2 net used on the same ships.

**Fig. 11:**
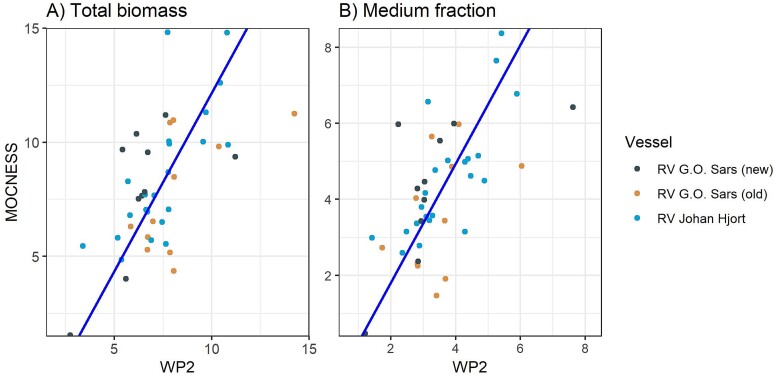
Annual autumn mean biomass values (g dry weight m^−2^) collected with MOCNESS versus WP-2 nets for three research vessels for (A) total biomass and (B) biomass of the medium size fraction.

The annual mean values by ships for total biomass and biomass of the medium fraction showed positive and significant correlations for MOCNESS versus WP-2 (Fig. 11; *r* = 0.40 and 0.41, *P* < 0.01). The regression slope (using the mixed axis regression model) was similar for total biomass and biomass of the medium fraction (1.33 and 1.34, respectively).

The time series were split into two parts (1992–2002 and 2003–2015), corresponding to the operation of the old and new G.O. Sars’s. The relative biomass distribution among the three fractions was quite similar in the pairwise comparison of ships, with a tendency that the two G.O. Sars’s collected more of the small fraction and less of the large and medium fractions (Table V). This difference could be real, reflecting spatial differences in the operation of the ships (see Skjoldal *et al*., 2022 for spatial patterns in zooplankton biomass distribution). The decrease in the large fraction between the first and second periods reflects a decrease in this fraction after ~2005 (Skjoldal *et al*., 2022). The decrease of the large fraction was associated with an increase in the proportion of the medium fraction (Table V).

Compared to WP-2, MOCNESS collected more total biomass for three of the ship/period data sets (by 14–22%; negative by 6% in the fourth case; Table V). MOCNESS collected more of the large fraction compared to WP-2 in all 4 cases (by 30–53%) and more also of the medium fraction in all cases (by 3–29%; Table V). MOCNESS collected less of the small fraction in three of the four cases (by 17–32%, equal amount in the fourth case). The data for the two periods for RV Johan Hjort were nearly identical (Table V). The largest discrepancy for the difference between MOCNESS and WP-2 is for the comparison between the old G.O. Sars and Johan Hjort in the first period (Table V).

### MOCNESS performance on the ships

**Fig. 12:**
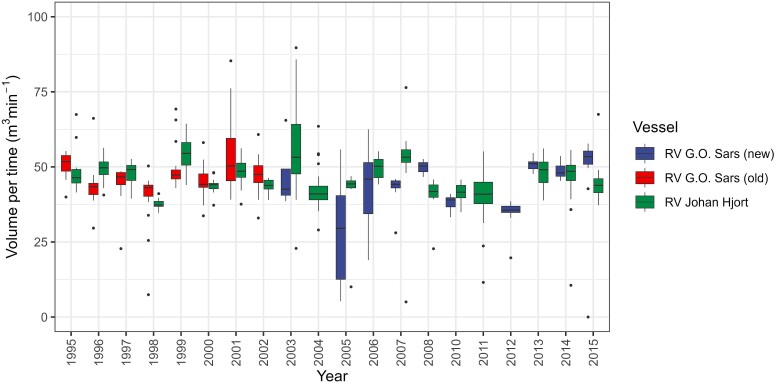
Annual values of volume filtered per unit time for MOCNESS profiles (minus upper net) for three research vessels from 1995 to 2015. Box-whisker plots show median (horizontal line), 25–75% quantiles (box), 5–95% quantiles (vertical line), and statistical outliers (dots). The *y*-axis has been cut at 100 m^3^ min^−1^, not showing three exceptional outliers.

Total volume filtered (minus the upper net, 25–0 m) and volume filtered per time (m^3^ min^−1^) showed relatively high values for the new G.O. Sars in 2013, 2014 and 2015, as well as for Johan Hjort in 2014 (Fig. S1). The high values corresponded to the use of flow factors ~6, following a calibration in 2013, and not factor 4.5, which had been used on most cruises prior to 2013 (see Table S1). When we rescaled the calculation of volume by using the common factor 4.5 for all ships and years, volumes filtered per time were nearly identical in comparisons of the three ships in terms of mean values (46.6, 46.7 and 46.9 m^3^ min^−1^ for Johan Hjort and the old and new G.O. Sars, respectively) as well as for the statistical distributions that were close to normal with median values close to the means (Table S4). Volume filtered per unit time for the three vessels over the series with oblique tows from 1995 to 2015 showed no trend (Fig. 12). A statistical model with “year” and “ship” as explanatory variables gave no significant effect of year, but a significant (*P* < 0.001) although low effect of ship, with only 2% of the total variance explained (*R*^2^ = 0.02).

## DISCUSSION

### Sampling performance of MOCNESS and WP-2 nets

Our results show a clear pattern of differences between MOCNESS and WP-2 that is consistent with what has been found earlier (Gjøsæter *et al*., 2000; Skjoldal *et al*., 2013). MOCNESS sampled more of the large size fraction (>2 mm) and less of the small fraction (<1 mm; note that these are screen sizes and not the size of individual zooplankton directly; see Skjoldal, 2021). Skjoldal *et al*. (2013) addressed two main mechanisms that affect the sampling efficiency of zooplankton nets: avoidance of the net by larger individuals with good swimming ability and extrusion of small individuals through the meshes of the net. The retention of individuals depends primarily on the width of the organisms rather than length. The retention as a function of width follows a steep sigmoid curve (logistic equation model) with 50% retention at a width approximately equal to the square mesh size, and 5% and 95% retention at widths about one-third smaller and one-third larger than the mesh size, respectively (Nichols and Thompson, 1991; Skjoldal *et al*., 2013).

Extrusion is the likely cause for the lower efficiency of catching the small zooplankton size fraction (<1 mm) by MOCNESS compared to WP-2 (Gjøsæter *et al*., 2000; Skjoldal *et al*., 2013). MOCNESS is towed at a targeted ship speed of 1.5 knots, equivalent to 0.77 m s^−1^. The mean volume of water filtered per tow time (47 m^3^ min^−1^; Table S2) corresponds almost exactly to this targeted speed if one assumes 45^o^ for the sum of the two angles θ and φ (frame and tow angles, see Fig. S8). However, the volume filtered per time is rather insensitive to towing speed due to the positive effect of speed on θ and the inverse relationship between mouth area and speed (Fig. 2; see Supplementary part B). The data for the cruises in 2007 and 2008 suggested that the sum of the angles θ and φ was substantially higher than 45 (59–63, Table S8). The mean net speed was ~ 1.2 m s^−1^ for both ships, with a range from 0.7 to 1.6 m s^−1^. This corresponds to 1.4–3.2 knots (mean 2.3 knots), which is substantially higher than the targeted speed of 1.5 knots. It is also much higher (by a factor 2 or more) than the targeted speed of 0.5 m s^−1^ for a vertical tow with the WP-2 net. The capture efficiency has been found to be sensitive to towing speed. Thus, increasing the speed from 1.5 knots with MOCNESS to 3 knots with BIONESS (which is a similar type of opening and closing net system; Sameoto *et al*., 1980) led to substantially reduced catch of small zooplankton, equivalent to that of a near doubling in mesh size from 180 to 333 μm with MOCNESS (Skjoldal *et al*., 2013).

The higher catch with MOCNESS for the large size fraction (>2 mm) is likely due to greater avoidance by large zooplankton of the WP-2 net than of the MOCNESS. Avoidance of mobile forms has long been recognized as a major source of error in zooplankton sampling (Clutter and Anraku, 1968; Sameoto *et al*., 2000), and avoidance by larger forms such as krill and amphipods can be substantial even for the relatively large MOCNESS sampler (Skjoldal *et al*., 2013; Wiebe *et al*., 2013; Eriksen *et al*., 2016). Increasing the size and towing speed of nets have been used as a means to reduce the problem of avoidance, and it is therefore likely that avoidance is larger for the smaller and slower WP-2 net compared to MOCNESS.

On a relative scale (expressed relative to the catch with WP-2), MOCNESS collected ~50% more of the large fraction, while it collected ~20% less of the small fraction. The small fraction contains small copepods such as *Oithona*, *Pseudocalanus* and young copepodites (CI–CIII) of *Calanus* species (Skjoldal, 2021; Skjoldal and Aarflot, 2023).

We found MOCNESS to collect more biomass in the medium fraction (1–2 mm) compared to WP-2 both in terms of relative proportions (Figs 5 and 9) and absolute values (Fig. 4). The magnitude of this effect was ~20–25%. The pairwise comparisons with the “same stations” data set gave a 16% higher catch of the medium fraction in MOCNESS compared to WP-2, while the “all stations” data gave a 26% higher catch (Table III). The medium size fraction includes the older copepodite stages (CV and adults and ~50% of CIVs) of *C. finmarchicus* and *C. glacialis*, which are dominant biomass components in the Barents Sea (Aarflot *et al*., 2018; Skjoldal, 2021; Skjoldal and Aarflot, 2023). It is not clear why MOCNESS would catch more of these copepods that make up the medium fraction. Avoidance of the smaller and slower WP-2 net by *Calanus* is a potential mechanism, although it is generally assumed that avoidance by copepods of vertical nets is limited (Skjoldal *et al*., 2013). We will revisit this issue after having considered sampling variance and potential bias due to operational factors.

The opposing effects of a higher catch of large zooplankton (due to less avoidance) and a lower catch of small zooplankton (due to extrusion) in MOCNESS compared to WP-2 lead to a relatively small difference in the catch of total zooplankton biomass by the two gears. With a higher catch of the medium fraction also, MOCNESS collected 14% more total biomass than WP-2 for the “all stations” data set and 5% more for the “same stations” set. The two gears therefore collect quite similar total amounts of zooplankton, and any difference between them is difficult to demonstrate statistically due to high sampling variance (which is discussed below). Thus, it takes a large and long-term data set, such as the one we provide here, to demonstrate smaller differences in the sampling performance of zooplankton nets.

### Sampling variance

It is a common observation in zooplankton sampling programs that the coefficient of variation (CV = SD/mean) is high (Downing *et al*., 1987; Pace *et al*., 1991). In our data sets, the CVs were ~0.8 for the total biomass (Table III). This variance includes sampling variability as well as spatial and temporal variations in the data sets. The sampling area spans the Atlantic water domain in the southern Barents Sea and the Arctic water domain in the northern Barents Sea (dominated by *C. finmarchicus* and *C. glacialis*, respectively; Skjoldal and Rey, 1989; Melle and Skjoldal, 1998; Falk-Petersen *et al*., 2009; Aarflot *et al*., 2018). Temporal changes in zooplankton biomass have been documented in relation to climate and predation (Dalpadado *et al*., 2012, 2014, 2020; Stige *et al*., 2014; Skjoldal *et al*., 2022; Skjoldal, 2023). Therefore, the variance structure in our data sets is expected to be composite, reflecting the influence of several factors.

Sampling variance at single stations can be a large fraction of the total variance in data sets such as ours. We have not generally included variance of replicate hauls in the sampling program in the Barents Sea, taking one tow with WP-2 or MOCNESS at each station. A method study with replicate hauls with WP-2 and Juday nets gave relative sampling variance (CV values expressed as percentage) of ~25, 40 and 80% for the small, medium and large fractions, respectively, and ~30% for total biomass (Skjoldal *et al*., 2019). Similar values of typically 20–50% were found in the gear intercomparison study reported by Skjoldal *et al*. (2013). A general relationship between replicate sampling variance and numerical density of zooplankton gives CV values of ~0.5–0.6 for densities of 0.1–10 individuals L^−1^ (Downing *et al*., 1987; Pace *et al*., 1991).

In our pairwise comparison with the “same stations” data set, the biomasses collected with MOCNESS and WP-2 were positively correlated, but regressions explained only about one-third of the variance (Table IV, Fig. 8). Thus, about two-thirds of the variance was unexplained, and we interpret this to reflect within-station sampling variance. The fact that the variance of the difference between MOCNESS and WP-2 was similar to the variances of the data for the two gears separately (similar SD in Table III) suggests that replicate sampling variance was a major component of the total variance of the data set. It is beyond the scope of this paper to do a detailed analysis of the variance structure and the ecological causes and implications of it. We limit ourselves to note that patchiness in zooplankton distribution is a common feature that relates to sampling variance (Wiebe and Holland, 1968; Omori and Hamner, 1982). Patchiness occurs at different scales reflecting physical processes (e.g. Langmuir cells, fronts, etc.) and animal behavior (e.g. swarming, vertical distribution, predation) (Haury *et al*., 1978). Sampling variance is influenced by the scale of patchiness in relation to the scale of sampling (Wiebe, 1971). The larger MOCNESS with longer (oblique) tow and larger volume of water filtered (by a factor of ~15–20) may integrate across small-scale patchiness, thus reducing variance compared to the smaller WP-2. In contrast, MOCNESS may reach larger-scale patchiness by having a higher probability of hitting large aggregations of zooplankton, thus causing increased sampling variance compared to WP-2.

The variance structure in our data sets was quite similar for the MOCNESS and WP-2 biomasses. This was suggested by similar CV values (Table III) and similar frequency distributions across all stations for the two gears (Fig. 6). Thus, while they sample at somewhat different scales, MOCNESS and WP-2 appear to be affected by (and reflect) similar processes and features that generate broadly similar variance in the data.

### Comparison of total biomass

Our comparison shows that MOCNESS and WP-2 gave similar but not the same results for zooplankton biomass. The outcome of a comparison would obviously reflect the composition of the zooplankton. MOCNESS would collect more total biomass if large forms predominated, whereas WP-2 would collect more if small forms were dominant. In our study, with autumn data from the Barents Sea where *Calanus* species are dominant in terms of biomass (Aarflot *et al*., 2018; Skjoldal and Aarflot, 2023), MOCNESS and WP-2 gave mean values of biomass that differed by 14% for the total data set (all stations) and by 5% for the pairwise comparison at the same stations. We note that to determine a statistically significant difference of 10% with measurements with a relative SD (CV) of 0.8 (see Table III), a minimum sample size of ~250 is required [confidence interval (0.1) = ~2 * SE, SE = SD/√*n*].

The difference between MOCNESS and WP-2 was consistent over time and space in showing similar temporal changes and geographical patterns for size fractions (Figs S4 and S5). There have been changes in the relative ratios of the zooplankton fractions in the ecosystem, reflecting climate variability and change as well as predation impacts from pelagic fish and other consumers of the food web (Dalpadado *et al*., 2014; Stige *et al*., 2014; Eriksen *et al*., 2017; Skjoldal *et al*., 2022; Skjoldal, 2023; Skjoldal and Sperfeld, 2024). The ecological changes are beyond the scope of the current paper, but they provide the motivation for why we are doing this methodological comparison.

The fact that MOCNESS and WP-2 gave very similar overall means for total biomass in the pairwise comparisons over the same stations (Table III) was probably coincidental, considering the wide scatter and variation among years in the difference between the two gears (Figs 7 and 8). By comparison, the larger “all stations” data set had higher total biomass for MOCNESS compared to WP-2 (by 14%, Table III), despite very similar proportions of the fractions in the two data sets (Fig. 5). As can be seen from Fig. 10B, this reflected basically more years with positive differences (for MOCNESS minus WP-2) over the time series with data for the three research vessels separately. The frequency distribution for the difference based on the annual mean values for the ships was close to normal (skewness and kurtosis were both 0.4) and had a median value of +0.6 g dw m^−2^, representing a difference of 8% compared to the total biomass collected by WP-2.

### Potential sampling bias due to calibration and ship operations

The speed of the MOCNESS sampler through water reflects the combination of ship speed and winch speed when the MOCNESS is towed obliquely from the lower sampling depth toward the surface. The MOCNESS is designed to be towed at an angle (θ) of 45^o^ to the vertical for the frame that holds the nets (with a rectangular opening). At this angle, the projection of the height of the rectangular opening frame (1.4 m) is 1.0 m in the vertical (cosine of θ = 45^o^), and the area of the net mouth opening is 1.0 m^2^ for an idealized horizontal haul. If towed with a lower angle (more vertical), the net opening is larger, and, if towed with a higher angle (flatter), the net opening is smaller than 1.0 m^2^. When towed obliquely, the filtered column of water (representing the trajectory of the MOCNESS nets) is tilted upward with angle φ to the horizontal. The height of this column is reduced relative to the vertical, and the effective opening of the net (height) is the cosine function of the sum of the two angles, θ and φ (Fig. S8). This can be illustrated by the case where the net is brought up obliquely with 45^o^ angle and the frame is hanging with 45^o^ angle in the vertical. In this case, the net is hauled flat along the oblique trajectory, and there is no effective opening (cosine of 45+45^o^ = 0). The effective opening of the MOCNESS nets is calculated in the algorithm for calculating filtered volume in the MOCNESS program according to equations in Wiebe *et al*. (1985) (see Supplementary part B).

The results related to the performance of MOCNESS operated on the three research vessels showed a clear difference in vertical hauling speed among the three ships, while the calculated filtered volume per unit time was not different when using a constant flow factor (4.5 m count^−1^) across the entire data set (Fig. 1, Tables S4 and S5). The change in flow factor to ~ 6 in 2013 gave higher volume filtered and a shift-up as a discontinuity in calculated volume filtered per time (see Fig. S1), and it was also associated with a reduction to negative values for the MOCNESS–WP-2 difference for the new G.O. Sars (Fig. 10B). Using flow factor 4.5 brought the values of volume filtered down to the same level as the rest of the series (Fig. 12). With the recalculated volume data with constant flow factor (4.5), there was no significant trend over the time series of the volume filtered per time, nor were there clear differences among the ships. This suggests that there has not been a shift or drift (gradual change) in the performance of the flowmeters, nor in the operation of the MOCNESS on the ships. The consistency over time is seen in the time series for RV Johan Hjort between 1992 and 2015. While there was some difference in vertical speed (Fig. 1B), with faster hauling (winch speed) on the new G.O. Sars by ~30%, this apparently did not cause a difference in the filtration performance of the MOCNESS, indicated by the similar volume filtered per time (Fig. 1A, Table S4).

In retrospect, the use of a constant flow factor gives a consistent data set over the whole time series of operation of MOCNESS on the IMR research vessels. The shift in flow factor by 33% (from 4.5 to ~6.0 m per count) in 2013 does not seem warranted as it caused a step change in volume filtered per time. However, this is not to say that 4.5 is the correct factor to use. *In situ* calibration of the flowmeter mounted on a MOCNESS towed horizontally over a one nautical mile distance (in both directions to account for water currents) in 2013 as well as later calibrations have given flow factors ~6.0 (Table S1, Supplementary B; Strand *et al*., 2022). The factor 4.5 m count^−1^ was given in the manufacturer’s operation and maintenance instruction manual (Anonymous, 2003) and stems from early calibrations of the TSK flowmeter used on MOCNESS (Peter Wiebe, personal communication). We have found no calibration results that verify this lower factor of 4.5. Thus, 6.0 seems the appropriate factor to be used for the whole time series of MOCNESS results from the IMR vessels.

**Fig. 13:**
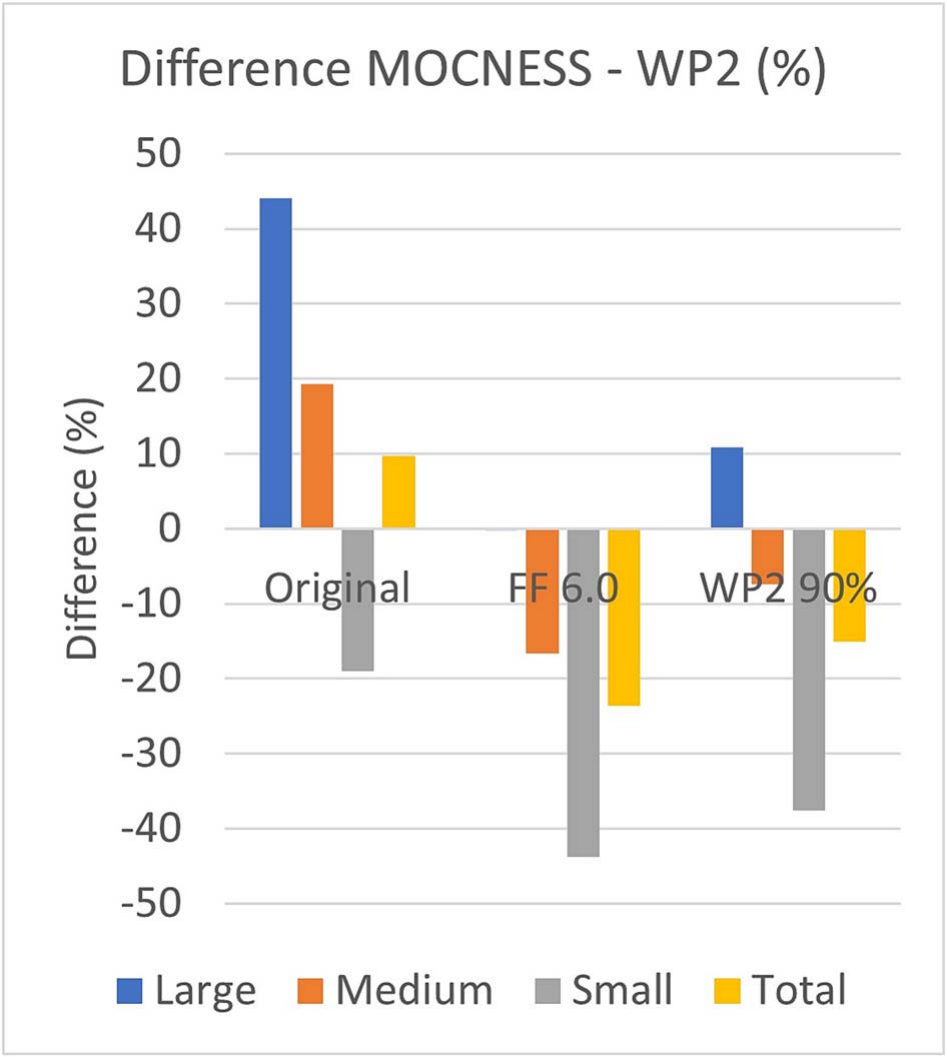
Difference in zooplankton biomass for samples collected with MOCNESS and WP-2 (MOCNESS minus WP-2, expressed as % relative to WP-2) for the data sets calculated with flow factors according to Table S1 (mostly 4.5; labeled Original), recalculated with flow factor 6.0 (FF 6.0), and recalculated with flow factor 6.0 and 10% reduction of the WP-2 results (WP-2 90%). Results are based on the annual mean values for the research vessels (shown in Fig. 10 for total biomass and in Fig. S7 for the medium fraction and total biomass). Note that the value for the large fraction in the FF 6.0 case is close to zero and not visible on the figure.

A higher flow factor (6.0 versus 4.5) means higher calculated volume (by factor 6/4.5) and lower biomass per volume (by factor 4.5/6) from direct proportionality. In addition, there is a factor 1.1 from the effect of flow factor on the calculated tow angle φ (see section MOCNESS performance in Methods and Supplementary part B, Fig. S14). We have adjusted the series of annual mean MOCNESS biomass values by research vessels to a constant flow factor of 6.0 across the series (1992–2015). This reduced the estimated biomass by a scaling factor (1/1.47 = 0.68) for the annual autumn data where flow factor 4.5 had been used (see Table S1), for each of the three fractions and total biomass. Relative to the WP-2 catch, the higher MOCNESS biomass of the large fraction was reduced from ~45 to ~0%, while the lower catch of the small fraction was increased from ~20% to over 40% (Fig. 13). For total zooplankton biomass, the results changed from MOCNESS collecting ~ 10% more to 24% less compared to WP-2.

The medium size fraction can be used as a performance indicator in the comparison between MOCNESS and WP-2 since it is not likely to be affected by extrusion (like the small fraction is) and probably less affected by avoidance compared to the large fraction. The medium fraction showed a similar normal-like distribution for the MOCNESS–WP-2 difference for the annual data (skewness 0.2 and kurtosis 0.1) to that shown for the total biomass, with a similar median value of 0.68 g dw m^−2^. This constitutes 19% compared to the mean biomass of the medium fraction collected by WP-2 (Fig. 13). Correcting the data to flow factor 6.0 shifted the results for the medium fraction down so that MOCNESS collected 16% less biomass than WP-2.

When comparing the results for MOCNESS to those for WP-2, we must also consider possible sources of error for the latter net. One effect is the smaller sampling gap to the bottom for WP-2 than for MOCNESS (9 m versus 25 m; Table II). This is due to a precautionary measure at routine cruises to have a safety margin of ~20–30 m between the MOCNESS and the bottom. The difference represents 6% of the height of the water column for an average water depth of 266 m at the MOCNESS sampling stations (Table S2). If biomass is concentrated in the deeper part of the water column, which is the case in the autumn situation (Aarflot *et al*., 2019), the correction due to under-sampling by MOCNESS would be larger. If we assume a 10% higher catch by WP-2 due to sampling closer to the seafloor, the MOCNESS would have collected ~ 10% more of the large fraction and nearly 40% less of the small fraction compared to WP-2 (Fig. 13). For the medium fraction, MOCNESS would still collect less biomass (by 7%) than WP-2.

The modified TSK flowmeter is mounted on top of the MOCNESS frame at an angle of 45^o^ so that it faces horizontally when the frame is towed at a tilt angle (θ) of 45^o^ (Wiebe *et al*., 1985). The orientation of the flowmeter relative to the tow direction for oblique hauls is given as the difference between the tow-angle φ and the angle (45-θ) for the orientation of the flowmeter relative to the horizontal (Fig. S8B). The effect of the off-axis orientation of the flowmeter on the flow counts is assumed to be a function of the cosine of the off-axis angle [φ − (45 − θ)]. *In situ* calibration of the flowmeter mounted on MOCNESS is normally done with horizontal tows over a known distance, while the MOCNESS is routinely used with oblique tows. Whether this difference has any effect on the flow factor derived from calibrations and used to calculate volume filtered is not known. This is an issue that should be examined further. It is relevant also for other sampling gears such as MultiNet, which can be towed horizontally or obliquely.

IMR decided in 2018 to replace MOCNESS with MultiNet Mammoth sampler for routine use on IMR research vessels. On the transition, a comparison study was carried out that showed the two gears to give similar (not statistically different) results (Strand *et al*., 2022). The flowmeters on MultiNet come precalibrated from the manufacturer, and volumes filtered are calculated by algorithms that are not open source. Strand *et al*. (2022) simulated calibration by towing MultiNet Mammoth with three nets open, each over 0.5 nautical miles, and this repeated in the opposite direction. The results for volume filtered per nautical mile showed a CV (SD/mean) of ~5% for the results in each direction and with a difference of 35% between the two directions. This illustrates that calibration results come with associated variance and uncertainty.

## CONCLUSION

MOCNESS and WP-2 plankton samplers have been used in combination in routine monitoring of zooplankton in the Barents Sea since the late 1980s (Skjoldal, 2023). This has provided a large data set of ~900 MOCNESS profiles and nearly 3000 WP-2 samples with depth-integrated zooplankton biomass (g dry weight m^−2^) in three size fractions and total (sum of the fractions), which allows a detailed comparison of the two gears. Equipped with nets of the same mesh size (180 μm), MOCNESS and WP-2 gave broadly comparable results for total zooplankton biomass but with a clear and consistent difference with MOCNESS collecting less of the small fraction (<1 mm, by ~30%) and more of the large fraction (>2 mm, by ~20%) compared to WP-2. The effect on total biomass would depend on the plankton composition, with MOCNESS collecting more when large zooplankton predominate and less when small forms predominate.

High zooplankton sampling variance, which is inherent and reflects the nature of patchy zooplankton distribution, makes it generally difficult to reveal effects that are small and subtle. The two gears are operated by various crew and scientific staff on board the IMR research vessels, which could be a source of error and variance. However, sampling and analysis follow written procedures in a zooplankton handbook or manual that is part of the IMR quality assurance system (Hassel *et al*., 2020). The methods and procedures have been used at IMR with small modifications since the 1980s (Skjoldal, 2023). Much of the data collection has been done by a small group of skilled and dedicated technicians, which also is an assurance for methodological consistency. While there is evidence for a ship effect by three different research vessels in vertical hauling speed (winch speed) of the MOCNESS, the results on volume of water filtered per unit time (cubic meters per minute) were not different among the ships, nor with time over the series from 1992 to 2015.

A flow factor (in units of meters per count) is used to determine the volume filtered (and biomass per volume and integrated per square meter) for MOCNESS. Calibrations of the flowmeter used on MOCNESS in the last 10 years have given a factor of ~6.0, while most of the earlier results from the late 1980s have been obtained by using a factor 4.5. The use of different factors does not seem warranted from the consistency of volume filtered per unit time over the series. The results for the difference between MOCNESS and WP-2 in pairwise comparisons revealed no clear trend over time, which further suggests that there has not been any discernible “drift” due to changes in equipment or procedures (including the algorithm for calculating MOCNESS results). In retrospect, the flow factor 6.0 (m per count) appears to be the most appropriate, with factor 4.5 giving too low volume and correspondingly too high biomass (by 25% compared to factor 6.0). However, the issue of calibration of flowmeters mounted on sampling gears towed obliquely should be examined further by empirical studies, both *in situ* and in laboratory conditions.

## Supplementary Material

Supplementary_Part_A_031124_fbae065

Supplementary_Part_B_031124_fbae065

## Data Availability

The primary data for this paper are stored in the data base at the Institute of Marine Research in Norway, Norwegian Marine Data Centre.
