# Supplementary material for: Comparison of WP-2 and MOCNESS plankton samplers for measuring zooplankton biomass in the Barents Sea ecosystem
Source: J Plankton Res. 2024 Nov 28;46(6):654–72. doi: 10.1093/plankt/fbae065 (PMC11629780; doi:10.1093/plankt/fbae065)
Supplement: Supplementary_Part_A_031124_fbae065 [file supplementary_part_a_031124_fbae065.pdf]

Comparison of WP-2 and MOCNESS plankton samplers for measuring zooplankton biomass in the Barents Sea ecosystem

Hein Rune Skjoldal, Johanna Myrseth Aarflot, Tor Knutsen, and Peter H. Wiebe

Supplementary material

Part A – Additional tables and figures

Tables S-1 – S-5

Figures S-1 – S-7

Tables S-6 – S-8 and Figures S-8 – S-15 are found in
Supplementary Part B – Use and operation of MOCNESS at IMR

Table S-1. Flow factor (FF, m $count^{-1}$) used to calculate filtered volume for MOCNESS tows on autumn cruises in the Barents Sea with RVs Johan Hjort (1991-2015), G.O. Sars, old vessel (1987-2002), and G.O. Sars, new vessel (2005-2015). na – information on FF not available (assumed to be 4.5).

Year	Johan Hjort	G.O. Sars Old	G.O. Sars New	Other
1987 [4)]	No cruise	6.3		
1988 [4)]	No cruise	4.5		4.5 [1)]
1989 [4)]	No cruise	4.5		?5.63 [1)]
1990 [4)]	No cruise	na		na [1)]
1991 [4)]	na	na		
1992	4.5	na		
1993	4.5	4.5		
1994	4.5	4.5/2.3		
1995	4.5	4.5		
1996	4.5	4.5		
1997	4.5	4.5		
1998	4.5	4.5		
1999	4.5	4.5		
2000	4.5	4.5		
2001	4.5	4.5 [2)]		
2002	4.5	4.5		
2003	4.5		No MOC	
2004	4.5		No cruise	
2005	4.5		4.7 [3)]	
2006	4.5		4.7 [3)]	
2007	4.5		4.71	
2008	4.5		4.71	
2009	No MOC		No cruise	
2010	4.0		4.0	
2011	4.0		No cruise	
2012	4.0		4.0	
2013	4.0		6.154	
2014	4.0, 6.0		5.9	
2015	4.3		6.0	
2016	No MOC		No cruise	

[1)] – RV Eldjarn
[2)] – Corrected volume, divided by 2 due to double counts
[3)] – Derived from other RV G.O. Sars cruises the same year
[4)] – Net mesh size 333 µm

Table S-2. Summary statistics of variables related to operation of MOCNESS with oblique tows at autumn cruises in the Barents Sea, 1995-2015.

	Depth (m)	Sampling depth (m)	Volume (m^3)	Time (min)	Volume (m^3 min^{-1})	Vertical velocity (m min^{-1})
Mean	266	242	720	15.4	46.7	14.8
Median	267	243	659	15	45.4	14.3
Standard Deviation	88	86	348	6.8	7.6	4.5
Kurtosis	-0.27	-0.24	1.48	0.95	0.99	0.15
Skewness	0.12	0.22	1.00	0.82	0.98	0.54
Minimum	60	38	52	1	30.3	5.0
Maximum	490	468	2233	43	69.9	30.7
n	761	761	753	673	673	673

Depth is water depth (m) at sampling station, while sampling depth is the lower depth for the lower net in a profile. Volume and time are the total volume filtered and tow time, respectively, for all nets in a profile minus the upper net (usually 25-0 m). The next column is volume per unit time. Vertical velocity is calculated from the sampling depth minus 25 m (for upper net) divided by time. The number of observations (n = 685) for time and the derived variables, volume per time and vertical velocity, has been reduced by removing ~4.5 % of the lowest and highest volumes per time (keeping values between 30 and 70 m^3 min^{-1}) due to suspected errors in some of the recorded low and high tow times.

Table S-3. Correlation coefficients (Pearson r) (A) between operational parameters for MOCNESS used on autumn cruises in the Barents Sea, and (B) between operational parameters and zooplankton biomass in three size fractions and total. Results are for the 'all stations' data set. See legend to Table S-2 for more information.

	Depth (m)	Sampling depth (m)	Volume (m^3)	Time (min)	Volume (m^3 min^{-1})	Vertical velocity (m min^{-1})
A. Operational parameters						
Depth	1.00					
Sampling depth	0.97	1.00				
Volume	0.69	0.71	1.00			
Time	0.71	0.73	0.94	1.00		
Volume per min	0.03	0.04	0.28	-0.05	1.00	
Vertical (m min^{-1})	0.30	0.30	-0.31	-0.37	0.14	1.00
B. Biomass						
Large	0.29	0.32	0.24	0.30	-0.09	-0.01
Medium	0.32	0.34	0.23	0.28	-0.07	0.08
Small	0.23	0.24	0.15	0.20	-0.13	0.07
Total	0.35	0.38	0.26	0.32	-0.10	0.07

Table S-4. Volume filtered per unit time ($m^3 min^{-1}$) for MOCNESS profiles (minus upper net). Statistical summaries for each of the three research vessels between 1995 and 2015 when MOCNESS was operated with oblique tows. The lowest and highest ~4.5 % of values (<30 and >70 $m^3 min^{-1}$) have been excluded due to possible error in recorded times.

	Johan Hjort	G.O. Sars Old	G.O. Sars New
Mean	46.63	46.72	46.87
Median	45.20	45.85	44.46
Standard Deviation	7.54	5.74	10.49
Confidence Level (95 %)	0.77	0.80	2.04
Kurtosis	0.93	2.00	-0.39
Skewness	1.03	1.00	0.75
n	369	200	104

Table S-5. Vertical velocity ($m min^{-1}$) for oblique hauls of the MOCNESS plankton sampler for profiles minus upper net. Statistical summaries for each of the three research vessels between 1995 and 2015. The data set was reduced by removing profiles with the lowest and highest values of calculated volume filtered per unit time (<30 and >70 $m^3 min^{-1}$) due to possible error in recorded times.

	Johan Hjort	G.O. Sars Old	G.O. Sars New
Mean	14.58	12.73	19.84
Median	14.40	12.58	20.68
Standard Deviation	4.00	3.70	3.87
Confidence Level (95%)	0.41	0.52	0.75
Kurtosis	1.32	0.24	0.22
Skewness	0.73	0.53	-0.28
n	369	200	104

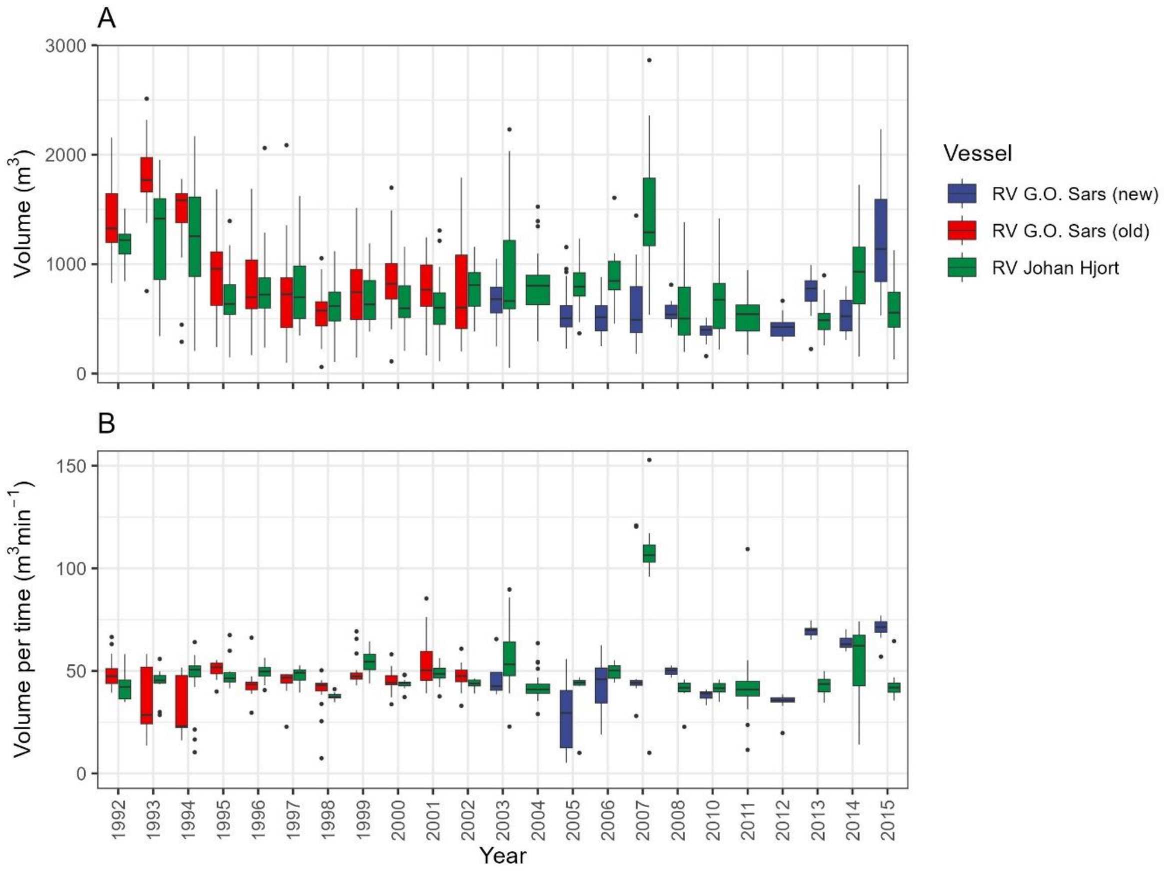

Fig. S-1. (A) Total volume filtered for MOCNESS profiles (minus upper net) and (B) volume filtered per unit time obtained by the three research vessels: Johan Hjort, 1992-2015; G.O. Sars, old vessel, 1992-2002; G.O. Sars, new vessel, 2003-2015. Box-whisker plots show median (horizontal bar), 25-75 percentiles (box), 5-95 percentiles (vertical line), and statistical outliers (dots). Higher volumes in 1992, 1993, and 1994 were due to stepwise hauls in contrast to oblique hauls from 1995 onwards. The high volume for Johan Hjort in 2007 is erroneous by factor 2 due to double count of the flowmeter (see section 'MOCNESS performance' in Methods).

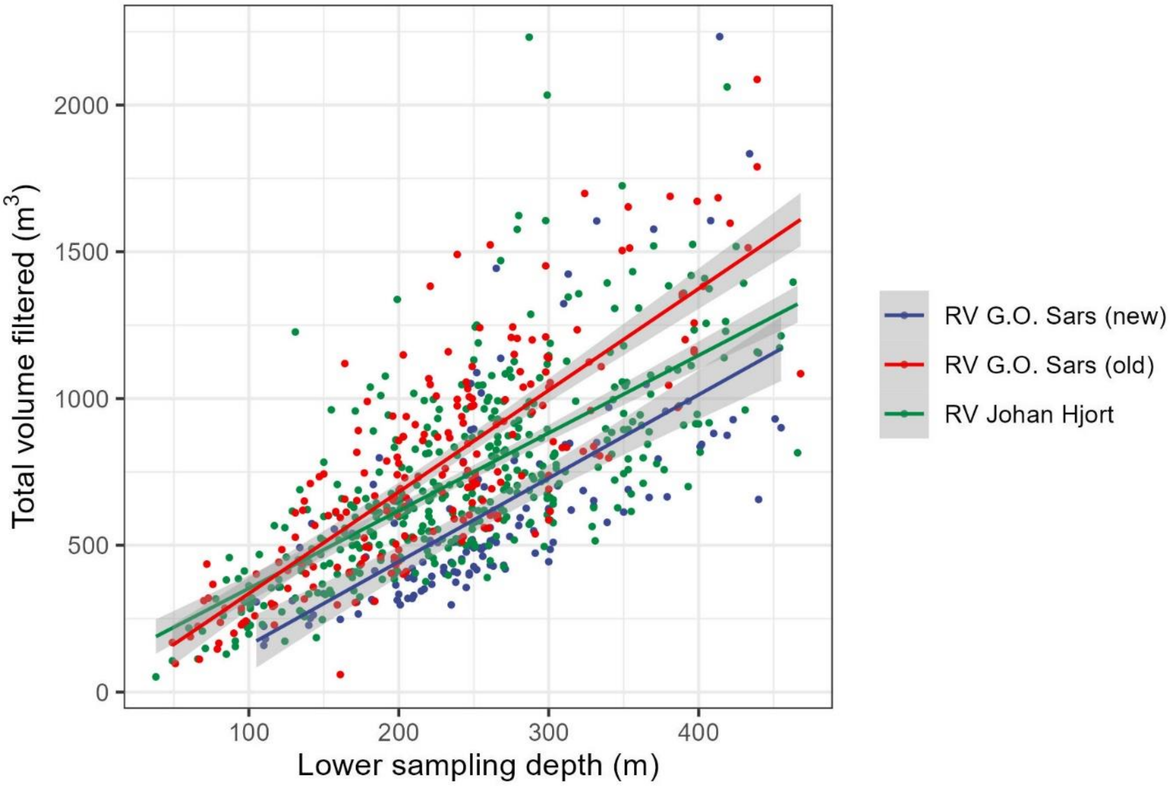

Fig. S-2. Scatter plots and regressions for the relationship between total volume filtered and lower sampling depth for MOCNESS profiles obtained with oblique tows (data for 1995-2015) for the three research vessels. The grey shaded areas are 95 % confidence bands around the regression lines.

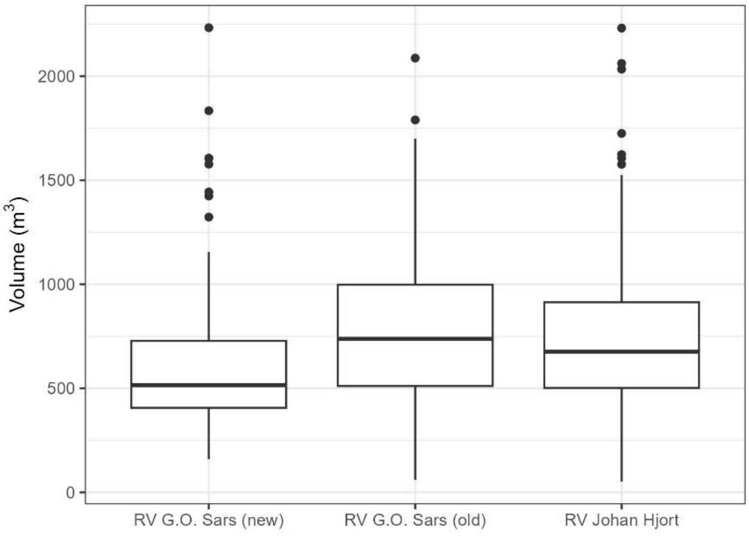

Fig. S-3. Volume filtered for MOCNESS profiles (minus upper net) obtained with oblique tows from the three research vessels, G.O. Sars (new) (2003-2015), G.O. Sars (old) (1995-2002), and Johan Hjort (1995-2015). Box-whisker plots show median (horizontal bar), 25-75 percentiles (box), 5-95 percentiles (vertical line), and statistical outliers (dots).

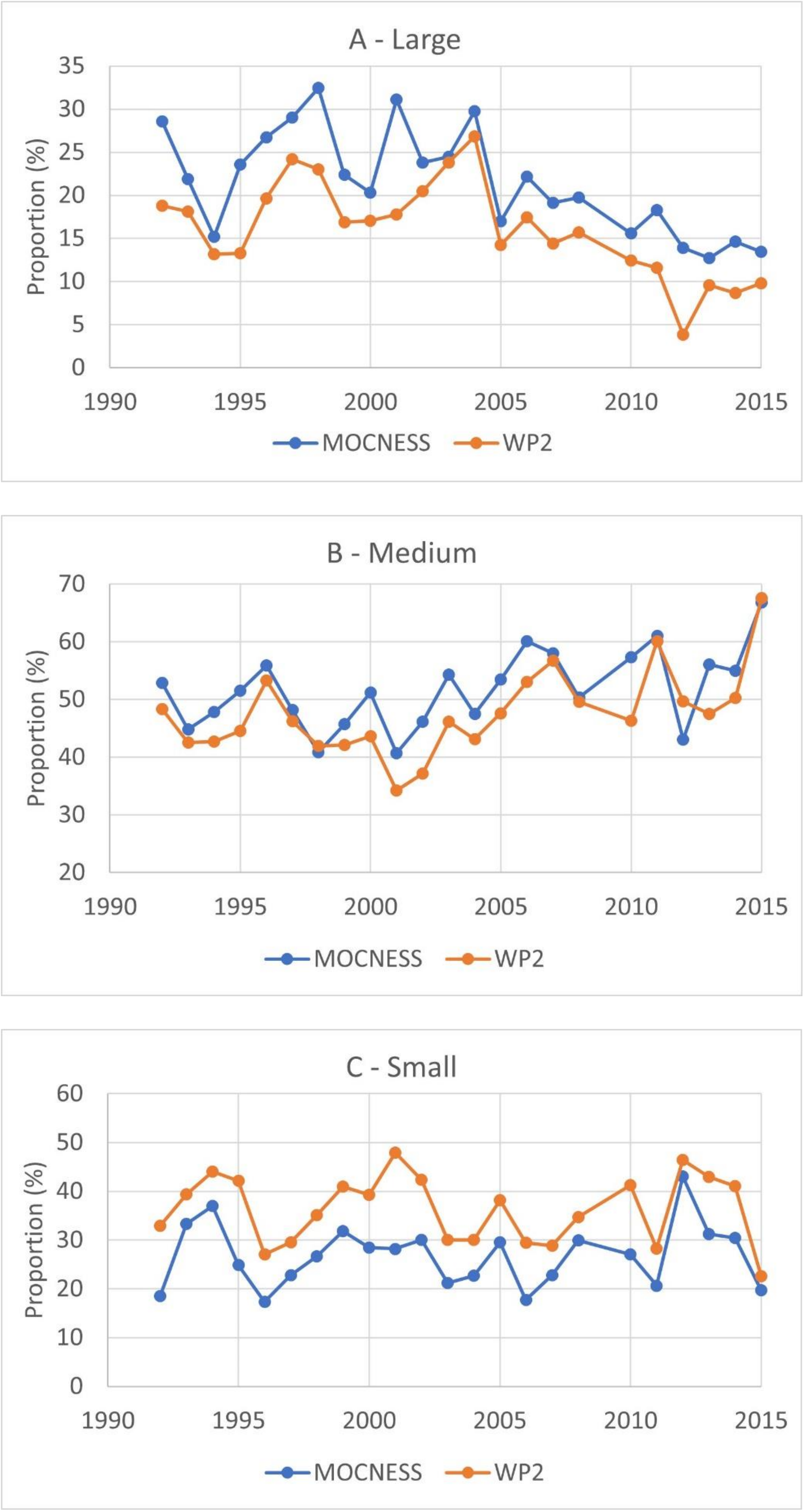

Fig. S-4. Proportion (%) of biomass collected in three size fractions obtained with MOCNESS and WP-2. Data are annual autumn means (across RVs) for the 'all stations' data set (see Table I). Note that data for 2009 are missing.

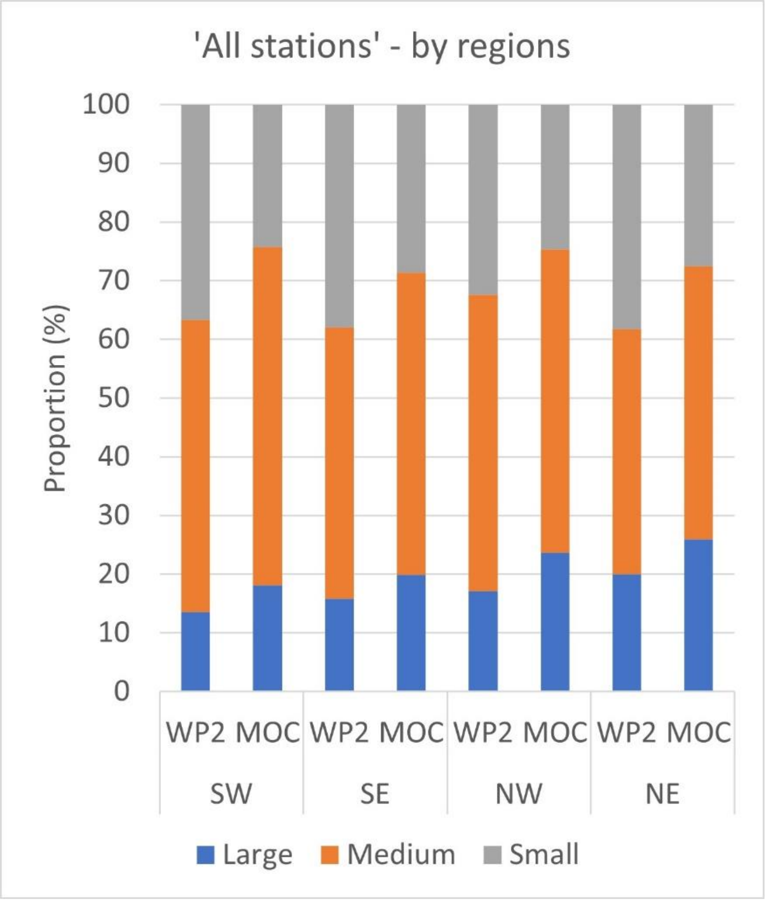

Fig. S-5. Proportion (%) of zooplankton biomass (g dw m^{-2}) in three size fractions (Large >2 mm, Medium 1-2 mm, Small <1 mm) as percentage of total biomass for results obtained with WP-2 and MOCNESS for four regions of the Barents Sea (SW: <74N and <30E; SE: <74N and >30E; NW: >74N and <30E; NE: >74N and >30E). Based on the 'all stations' data set, 1992-2015.

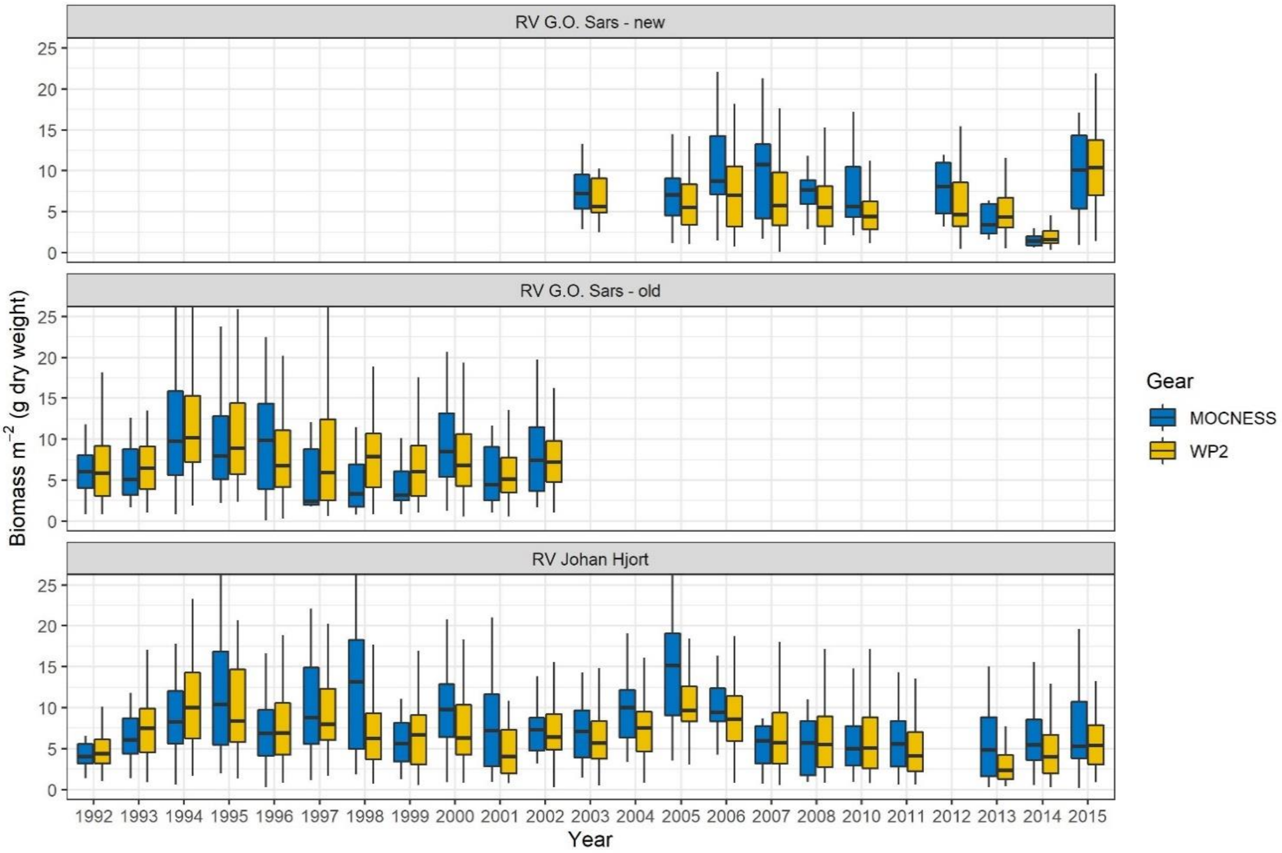

Fig. S-6. Box-whisker plots of annual total biomass values (g dw m^{-2}) for samples collected with WP-2 and MOCNESS from three research vessels: G.O. Sars, new vessel, G.O. Sars, old vessel, and Johan Hjort. Horizontal bars, boxes, and vertical lines show median values, 25-75 percentiles, and 5-95 percentiles, respectively. See Table I for number of samples for each year and ship.

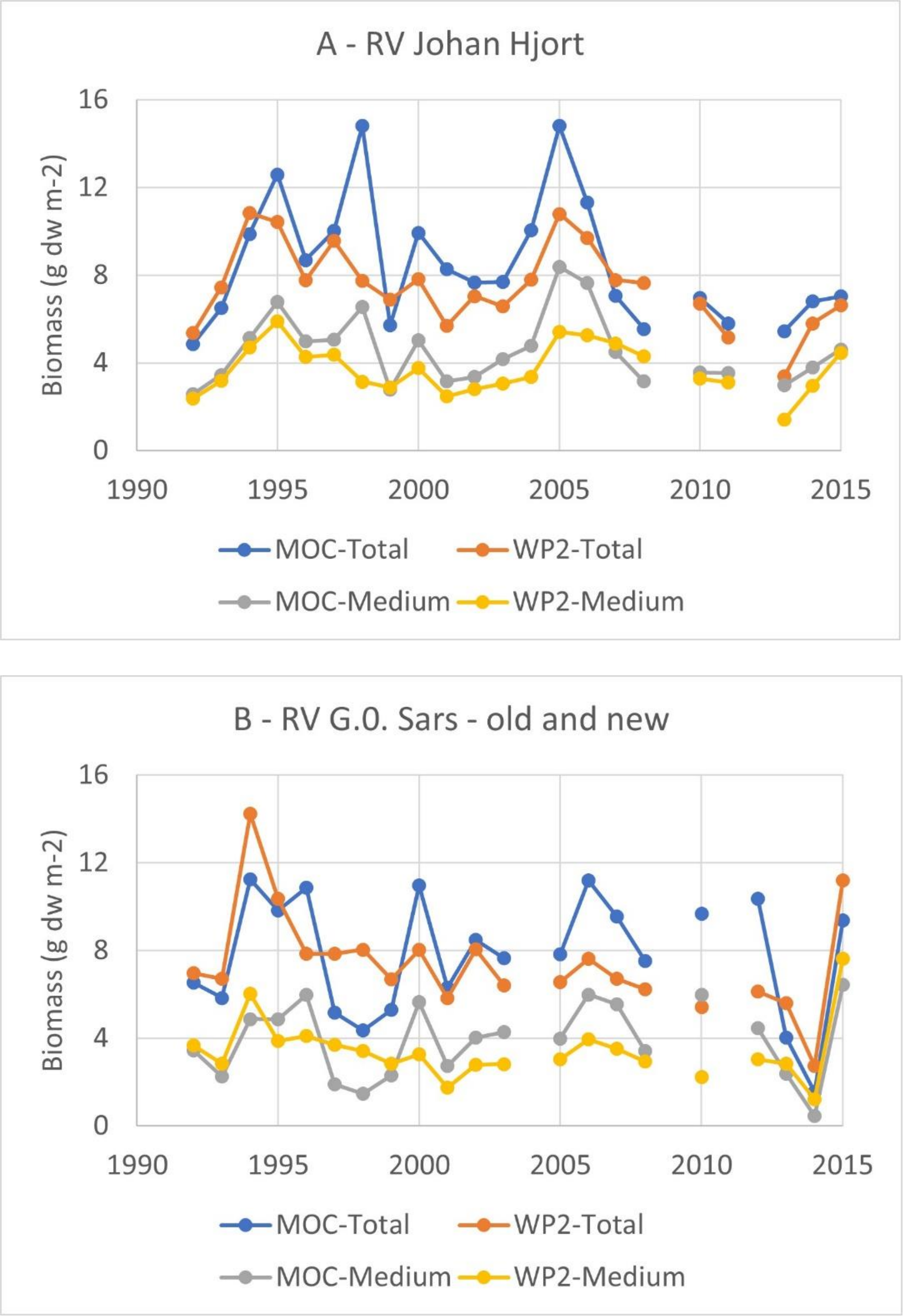

Fig. S-7. Annual autumn mean values of total zooplankton biomass (Total) and biomass of the medium fraction (Medium) obtained with MOCNESS (MOC) and WP-2 for (A) Johan Hjort, and (B) old (1992-2002) and new (2003-2015) G.O. Sars.
