# Supplementary material for: Comparison of WP-2 and MOCNESS plankton samplers for measuring zooplankton biomass in the Barents Sea ecosystem
Source: J Plankton Res. 2024 Nov 28;46(6):654–72. doi: 10.1093/plankt/fbae065 (PMC11629780; doi:10.1093/plankt/fbae065)
Supplement: Supplementary_Part_B_031124_fbae065 [file supplementary_part_b_031124_fbae065.pdf]

Comparison of WP2 and MOCNESS plankton samplers for measuring zooplankton biomass in the Barents Sea ecosystem

Hein Rune Skjoldal, Johanna M. Aarflot, Tor Knutsen, and Peter Wiebe

Supplementary material Part B

Use and operation of the MOCNESS plankton sampler by the Institute of Marine Research in the Barents Sea

MOCNESS units

The Institute of Marine Research (IMR) in Norway acquired its first MOCNESS 1-m^2 plankton sampler in the early 1980s, along with a MOCNESS 10-m^2 system for targeted sampling of zooplankton and fish larvae, respectively. A second 1-m^2 unit was bought in the late 1980s, while a third 1-m^2 MOCNESS unit was taken over from the University of Bergen in the early 1990s. Another 1-m^2 MOCNESS unit was ordered in 2002 for the new RV G.O. Sars that went into service in 2003. At this time, an additional 16-bits electronics package including a deck unit and PC was bought to make an already existing mechanical unit fully operational and replace an old 12-bit electronic system. The last complete MOCNESS system (mechanics and electronics), including a significant number of spare components, was bought in 2009.

A MOCNESS consists of two main parts: 1) the net system itself with sensors that are towed in the water behind the vessel, and 2) an electronic deck unit that receives and processes signal from the net system via a cable to the ship. The opening and closing nets are mounted on an elongated rectangular frame, designed to give a net opening of 1 m^2 (1 m x 1 m) when the frame is towed with an angle of 45 degrees to the vertical. The sensors on the underwater unit collect environmental and operational parameters. The operational sensors record pressure (depth), angle of the frame, speed through water recorded with a flowmeter, and release of net bars that signals closing/opening of nets.

Microprocessors in the underwater and deck units communicate via a cable that transmits signals from the ship for opening and closing of nets, and data from the underwater sensors to the deck unit for calculating and displaying results.

Use and calibration of flowmeters

A modified version of the Japanese TSK flowmeter (Tsurumi-Seiki-Kosakusho Co., Ltd.) has been used on the MOCNESS throughout its operation. The TSK is a mechanical flowmeter with a four-bladed impeller mounted inside a cylindrical housing, and with a magnetic switch that registers a count per rotation of the impeller. The flowmeter is mounted on top of the net-frame so that it faces horizontally when the frame is towed at an angle of 45 degrees. The flowmeter is calibrated with a flow factor that converts counts (per 4 seconds) to unit of m per second (net velocity through the water). The volume of water filtered through a net (assuming 100 % filtration efficiency) is calculated from the flow counts times flow factor and net mouth

opening (1 m width times height estimated from angle). The algorithm for calculating volume also includes the vertical velocity of the net and the angle of the tow (relative to the horizontal) as it is hauled obliquely through the water column toward the surface. More information is provided in the following section *Calculation of volume of water filtered*.

MOCNESS was operated with a flow factor value of 4.5 m per flow count on all cruises in the Barents Sea between 1988 and 2007 (Table S-1, in Supplementary Part A). This is a value given in the MOCNESS manual from the supplier (Anonymous, 2003, page 19). Peter Wiebe obtained a factor of 4.7 when calibrating TSK flowmeter from a small boat over a fixed distance in a harbor (Wiebe, pers. comm.). There appears to have been only a restricted number of calibrations of the specific flow meters used at IMR. Results from calibration events documented in logbooks from IMR cruises are summarized in Table S-6.

Table S-6. Calibration events of flowmeter on MOCNESS towed in both directions (result 1 and 2) over a fixed distance. Results are given in units of m per flow count, and the average value is the estimated flow factor from the calibration event.

Date	Ship	Location	Result 1	Result 2	Average
February 1990	Eldjarn	Barents Sea	5.86	7.04	6.6
15 August 1995	G.O. Sars	Balsfjord	5.7	5.35	5.53 [1)]
6 June 2013	G.O. Sars	North of Iceland	4.3	8.0	6.15
10 August 2014	Johan Hjort	Near Karlsøy, Troms	6.15	6.19	6.17
27 November 2014	Johan Hjort	Byfjorden, Bergen	5.29	6.36	5.83
29 April 2015	Johan Hjort	Eigerøya, Norwegian Trench	5.92	5.48	5.70
1 May 2015	Johan Hjort	Shetland Islands	5.30	6.29	5.79

1) Defect flowmeter with one of the four impeller blades missing.

A calibration in February 1990 gave a flow factor of 6.6 m per count. Another calibration in August 1995 with a defect flowmeter (missing one blade) gave a factor of 5.53 m per count. These calibration results were apparently not used to change the flow factor from 4.5, which was used consistently up to 2008 on RV Johan Hjort (Table S-1). On the new RV G.O. Sars, a slightly higher value of 4.71 m per count was used from 2005 to 2008.

In 2010-2012, a flow factor of 4 was used in calculations of volume filtered. It is not clear to us where this value comes from and why the flow factor was changed from 4.5 to 4.0. On a cruise with the new G.O. Sars in 2013 (on 6 June in waters north of Iceland), an *in situ* calibration was done by towing the MOCNESS at 50 m depth over a distance of 0.5 nautical

mile, in both directions. The result was a flow factor of 6.154 m count^{-1} (average of 4.3 and 8.0 for the two directions; Table S-6), which was used on the autumn cruise with G.O. Sars in the Barents Sea that year. Flow factor 4 was used on Johan Hjort in 2013. Two *in situ* calibrations were done on cruises with Johan Hjort in 2014 (10 August and 27 November). They gave flow factors of 6.2 (6.15 and 6.19 for the two legs) and 5.8 (5.29 and 6.36) m count^{-1}, respectively (Table S-6). A flow factor of 6 was used on Johan Hjort on the autumn cruise in 2014, while a factor of 4.3 was used in 2015 (we find no explanation for why the factor was changed back from 6 to near 4.5 in 2015). Flow factors of 5.9 and 6 were used on G.O. Sars in 2014 and 2015 (Table S-1).

Calculation of volume of water filtered

Data are transmitted every 4 seconds by the MOCNESS underwater instrument unit when the MOCNESS is operated in a haul. The primary (raw) data on pressure (depth), angle of the frame, flow counts, and opening and closing of nets are recorded and stored along with calculated results of speed through water (from the flow counts of the flowmeter), vertical velocity (from pressure), and volume filtered through a net. The volume filtered is estimated from flow counts multiplied by flow factor (m per count), net opening (estimated from angles), and vertical velocity, using equations detailed in Wiebe et al. (1985). The total volume filtered by a net is the integral volume in the time from opening to closing of the net.

There are three angles used when calculating volume filtered by a net:

- Angle θ (theta), which is the angle between the towed MOCNESS frame and the vertical (Fig. S-8A)
- Angle φ (phi), which is the tow angle for oblique tow relative to the horizontal (Fig. S-8A), and
- An angle for the offset of the flowmeter relative to the direction of the tow (Fig. S-8B)

θ is measured with an inclinometer mounted on the MOCNESS frame and recorded with the data stream every 4 seconds. φ is calculated from the net velocity (NV) in the oblique direction of the tow and the vertical velocity component estimated from recorded pressure (depth). Using Pythagoras, the vertical velocity is the sine function of φ multiplied by NV (the hypotenuse of the geometrical configuration; Fig. S-8A).

The effective mouth area of a MOCNESS net is determined by the projection of the frame onto a plane perpendicular to the oblique tow direction (Fig. S-8A). The height of the projection is given as the cosine function of the sum of angles θ and φ multiplied by 1.4 m, which is the height of the frame (Fig. S-8A).

The flowmeter is mounted on top of the frame at an angle of 45 degrees so that it faces in the horizontal direction when the frame is towed at angle θ of 45 degrees. The direction of the flowmeter relative to the horizontal is given by 45-θ (Fig. S-8B). If θ is <45 (the frame is more vertical), the flowmeter points upwards with a positive angle (45-θ > 0) relative to the horizontal. If θ is >45 (the frame is towed more horizontal), the flowmeter points downwards

with negative angle (45-θ < 0). The orientation of the flowmeter relative to the oblique tow-direction is given as the difference between φ and the angle 45-θ, which becomes φ+θ-45.

The off-axis orientation of the flowmeter relative to the tow direction (φ+θ-45) is included when filtered volume is calculated. The volume is found as the product of net velocity (flowmeter counts * flow factor) times the opening of the net in the direction of tow (cos (θ + φ)). For net velocity, the flowmeter counts are adjusted upwards by assuming the efficiency of the flowmeter being reduced as a cosine function of the off-axis orientation (φ+θ-45) (slower rotation proportional to cos (φ+θ-45)). The adjustment is done by dividing the flow counts by the cosine function.

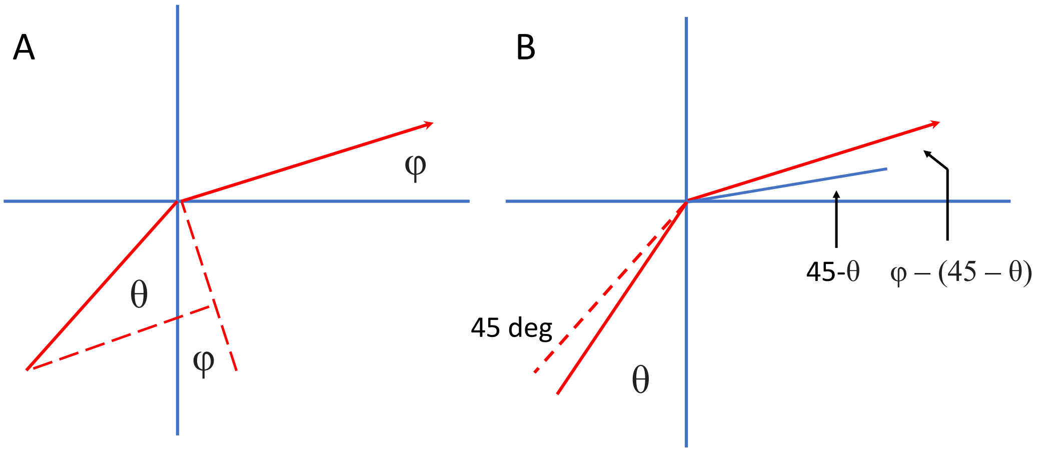

Fig. S-8. Geometry of MOCNESS towed obliquely through the water. A. The MOCNESS frame (height 1.4 m) is towed at an angle θ relative to the vertical, with the oblique tow direction given by angle φ relative to the horizontal. φ is also the angle between a line perpendicular to the tow direction and the vertical. The effective mouth area of the net is the projection of the frame onto the perpendicular plane, with the height given by cosine of the sum of θ and φ. B. Orientation of the flowmeter mounted on top of the frame at angle 45 degrees. The orientation of the flowmeter relative to the horizontal is angle 45 - θ. The orientation relative to the tow direction is the difference between φ and angle 45 – θ.

Program versions used to calculate volume filtered

The first MOCNESS units had 12-bit electronics systems, and custom-built computer programs based on BASIC or, from the mid 1980's, PASCAL programming language to acquire data from the underwater unit. The MOCNESS bought for the new RV G. O. Sars in 2003 used a 16-bit system (Anonymous, 2003) with a Visual Basic 3.0 program (MOCNESS.EXE) designed to run the electronics system for MOCNESS under WINDOWS 3.11 or WINDOWS 95. There were several releases and updates of this software with the

newest version run during a comparison exercise undertaken in 2019 with two multiple plankton samplers: MOCNESS and Multinet Mammoth (Strand et al., 2022).

We have checked the various program versions that were in use at IMR and found them to correctly include the equations for calculating filtered volume from Wiebe et al. (1985). In the early years (1980s and 90s) when using the 12-bit systems, the raw data was written to 1.44 MB floppy disks, which were stored with the logbooks from the individual cruises. Some of these data were at a later stage transferred to computer disks at IMR. As vessel computer facilities later evolved, data were transferred to servers onboard the vessels and subsequently to land-based servers when the ships returned to port. With the more recent 16-bit systems using the MOCNESS.EXE program (Visual Basic developed software), data were stored in three different files on computer: the *.RAW, *.PRO and *.TAB files. The content of the *.TAB files changed moderately over the years after feedback and needs identified by users.

Operation and maintenance

The maintenance of the MOCNESS equipment at IMR was shared by personnel from the plankton group and from the group that operated and serviced the scientific instruments used on the research vessels (such as echo sounders, CTDs, etc.). The plankton group generally took care of the nets, while the electronics were the responsibility of the engineers from the technical group.

Technical issues frequently occurred with malfunctioning of equipment due to harsh weather conditions and frequent use of our MOCNESS units. Mechanical wear and tear, including old and partly outdated hardware components in the deck- and underwater units, made maintenance difficult. Electronic units were occasionally sent back to the supplier in the USA for repair. However, spare parts like microprocessors, integrated circuit boards as well as other components were generally hard to acquire. The old units were kept going by taking spare parts from each other, until this predatory approach was no longer possible.

MOCNESS was used on two IMR research vessels on autumn cruises in the Barents Sea each year (see Table S1 in Supplementary Part A). Two functional MOCNESS units were thus needed, and this was achieved by combining equipment from the units available at IMR and occasionally from the University of Bergen. We have not been able to track information on the specific units (in situ and deck) used on the various cruises in the Barents Sea from which we report results. However, we assume they were well maintained and functional. In the logbooks from the cruises, there are notes on technical difficulties encountered with specific tows (stations), and how this was dealt with (e.g., if there was uncertainty about opening and closing of two nets, results may have been used to represent the depth interval for the two nets combined).

Data quality evaluation

The MOCNESS data which are stored in the IMR database, have been quality assured when they were entered. The quality assurance included recalculation of filtered volumes where

difficulties were noted during cruises. For some cruises in the 1990s and early 2000s, the recalculation was done by Tor Knutsen (one of the present authors) based on an in-house computer program for which results were verified and compared with results from other programs used at sea including the Visual Basic program MOCNESS.EXE that became available from approximately 2003 onwards. As part of the present study, we have done some additional quality evaluation of the MOCNESS data from the Barents Sea. Using MOCNESS raw data stored on floppy discs and local servers, we recalculated volume filtered for selected cruises where we suspected that some data could be erroneous. However, the recalculated volumes were found to agree closely with the processed data stored in the IMR database, deviating by at most a few percentage points. One reason why there can be some deviation lies in the way data, data spikes, or missing data due to transmission loss were handled in software during program execution at sea and during later re-computations that included use of the MOCNESS.EXE replay function.

The raw data from the MOCNESS profiles contain records of the frame angle θ every 4 seconds. The tow angle φ (see Fig. S-8) is calculated from flow counts (converted to net velocity) and vertical velocity (from recorded pressure) as part of the algorithm for calculating volume filtered during program execution. The summary tables provided as output with information for each net of a profile (depth interval, velocity, volume filtered) did not contain information on the two angles which are used in the calculations. We ran new computations for the two cruises with Johan Hjort and G.O. Sars (new) in 2007 and 2008 where we included θ and φ as output along with recorded flow counts and calculated velocities (net, horizontal, and vertical) and volume filtered. Results from this exercise are presented in a subsequent section.

Volume filtered per unit time

We used volume filtered per unit time (m^3 per min) as an initial performance indicator for the various MOCNESS tows. The sum of volumes for the various nets in a vertical profile was divided by the duration of the haul, excluding the upper net (usually 25-0 m) due to lack of recorded time for its closure. Time series as box-whisker plots of volume filtered per unit time are shown in Fig. S-1 (in Supplementary Part A), along with total volume filtered for the MOCNESS results obtained by the various research vessels from 1993 to 2015.

An issue of double counts

One of the underwater units apparently recorded two counts from the flowmeter for every rotation. This was due to a magnetic switch replacement that resulted in two counts registered and not one per propeller rotation. The issue is noted in some of the logbooks from cruises. On the cruise with G.O. Sars in 1994, a flow factor of 2.3 (2.25 rounded, being half the value 4.5) was used to account for the effect of double counts. For the cruise with GOS in 2001, the issue of double counts was noted, and the volumes calculated with the wrong flow factor 4.5 were divided by factor 2 after the cruise.

In our review of MOCNESS data, we noted that the volume filtered per time for the cruise with Johan Hjort in 2007 was about twice as high as for other cruises (see Fig. S-1). Inspection of the raw data revealed flow counts twice as high than usual (~2 counts per 4 seconds versus ~1 count per 4 seconds). We concluded from this that the data for Johan Hjort in 2007 were affected by double counts which had not been recognized in previous quality assurance. We corrected the results for Johan Hjort in 2007 by dividing the volumes by factor 2 and multiplying biomass values (per m^2, integrated from biomass values per m^3) by the same factor 2. The correction brought the volume filtered per time for this cruise in line with other cruises of the time series (see Fig. 12 in the main paper).

Vertical towing velocity

The vertical velocity for MOCNESS hauls were calculated from the lower depth of the deepest net (net 1) and the total time for a profile minus the upper net (maximum depth minus 25 m). The vertical velocity (expressed as m per min) was significantly higher for the new G.O. Sars (mean 19.8 m min^{-1}) compared to Johan Hjort (14.6 m min^{-1}) and the old G.O. Sars (12.7 m min^{-1}) (Table S-7).

Table S-7. Vertical velocity (m min^{-1}) for MOCNESS hauls as statistical summaries (mean, standard deviation, and standard error) for three research vessels over their times of operation with autumn cruises in the Barents Sea in the period 1995-2015. Values are calculated for the depth interval from lowest sampling depth minus the upper net (usually 25-0 m). n is number of MOCNESS profiles.

	Johan Hjort	G.O. Sars (Old)	G.O. Sars (New)
Mean	14.58	12.73	19.84
SD	4.00	3.70	3.87
SE	0.21	0.26	0.38
n	369	200	104

Time series as annual mean values of vertical velocity for the three vessels reveal variation around 15 (12-17) m min^{-1} with no clear trend for Johan Hjort (Fig. S-9). Relatively low values (10-11 m min^{-1}) were seen in the three first years (1995-97) for the old G.O. Sars. The values for the new G.O. Sars were consistently high (>20 m min^{-1}) in all but three years (2005, 2014, 2015), when they were similar to the values for Johan Hjort (~15 m min^{-1}) (Fig. S-9).

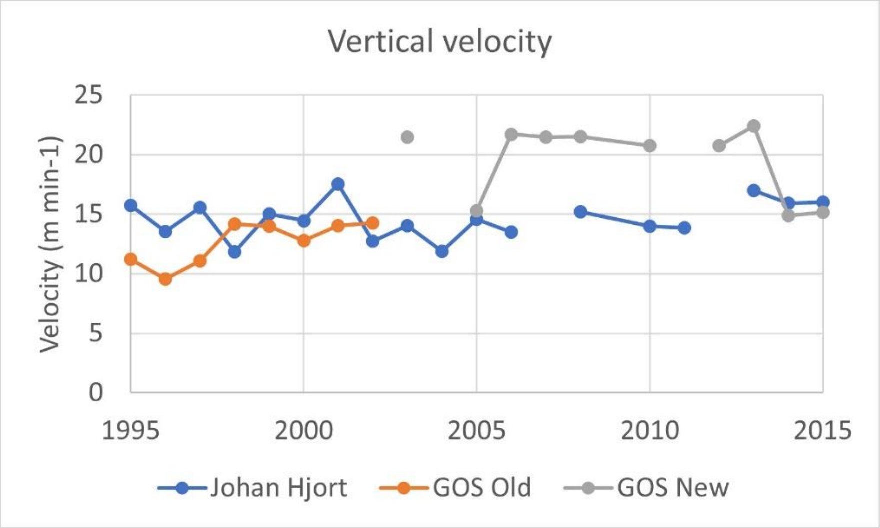

Fig. S-9. Time series of vertical velocity of MOCNESS hauls as annual mean values for the three research vessels. Values are calculated for the water column from the deepest sampling depth integrated for all nets except the upper one (usually 25-0 m).

Influence of towing speed on frame angle θ, net mouth area, and volume filtered – results from reprocessing of data from cruises in 2007 and 2008

Statistical summaries of the reprocessed data from the cruises with Johan Hjort and G.O. Sars in 2007 and 2008 are given in Table S-8. The reprocessing was done with the original flow factors of 4.5 (m per count) for Johan Hjort in 2008 and 4.7 for G.O. Sars in both years. The original data for Johan Hjort in 2007 was calculated with flow factor 4.5. Due to the issue of double counts, the reprocessing was done with flow factor 2.25. The distributions of the operational variables were close to normal, indicated by low values of skewness and excess kurtosis (<1 in most cases; Table S-8).

The frame angle θ varied around the targeted 45 degrees, with a range of 30-54 for G.O. Sars and 30-63 for Johan Hjort. θ was significantly higher for Johan Hjort compared to G.O. Sars (mean values of 47.7 and 51.9 versus 44.8 and 43.8), while the opposite was the case for the tow angle φ (12.3 and 10.6 versus 15.4 and 15.2) (Table S-8). φ and θ were inversely related for the data for the two vessels, with $R^2 = 0.42$ ($p < 0.001$) for the data for the two ships combined (Fig. S-10). The regression slope was -0.64 (geometric mean regression), reflecting that the amplitude of change was less for φ than for θ.

Table S-8. Statistical summary of the reprocessed MOCNESS data for the autumn cruises with RVs Johan Hjort and G.O. Sars in 2007 and 2008. Theta (θ) and phi (φ) are the frame and tow angles. Mouth area is the effective opening of the net (cosine function of θ + φ). Flow count is per 4-seconds. SD and SE are standard deviation and standard error. The data are for individual MOCNESS nets (n; excluding net 0 and the uppermost net (25-0 m)) from 18 and 11 (JH) and 21 and 13 (GOS) MOCNESS tows.

	Theta				Phi				Mouth Area (m^2)				Volume (m^3 min^{-1})			
	Johan Hjort		G.O. Sars		Johan Hjort		G.O. Sars		Johan Hjort		G.O. Sars		Johan Hjort		G.O. Sars	
	2007	2008	2007	2008	2007	2008	2007	2008	2007	2008	2007	2008	2007	2008	2007	2008
Mean	47.72	51.93	44.85	43.76	12.27	10.58	15.35	15.17	0.70	0.64	0.69	0.72	47.03	43.56	47.30	51.78
Median	48.67	52.74	44.84	43.18	11.68	10.49	15.61	16.15	0.69	0.64	0.68	0.71	47.06	43.54	47.39	51.41
SD	5.43	6.22	4.01	4.57	4.31	2.47	2.12	2.61	0.07	0.12	0.09	0.08	3.38	1.91	1.40	1.69
SE	0.58	0.98	0.40	0.56	0.46	0.39	0.21	0.32	0.01	0.02	0.01	0.01	0.36	0.30	0.14	0.21
CL (95%)	1.16	1.99	0.79	1.11	0.92	0.79	0.42	0.64	0.02	0.04	0.02	0.02	0.72	0.61	0.28	0.41
Minimum	30.40	40.40	33.72	30.18	4.06	6.61	6.88	8.43	0.56	0.41	0.55	0.61	35.71	39.89	41.91	49.36
Maximum	57.29	63.13	53.90	52.59	28.95	16.66	20.57	18.81	0.93	0.87	0.92	0.95	54.51	49.85	50.30	57.22
Kurtosis	0.82	-0.99	0.05	0.14	3.68	-0.39	2.93	-0.35	1.18	-0.85	0.07	0.45	0.30	2.04	1.71	0.44
Skewness	-0.86	-0.10	-0.40	0.02	1.40	0.37	-0.94	-0.83	0.90	0.00	0.73	0.83	-0.45	0.81	-0.57	0.78
n	87	40	101	67	87	40	101	67	87	40	101	67	87	40	101	67
	Flow counts (per 4 s)				Net speed (m s^{-1})				Horizontal speed (m s^{-1})				Vertical speed (m s^{-1})			
	Johan Hjort		G.O. Sars		Johan Hjort		G.O. Sars		Johan Hjort		G.O. Sars		Johan Hjort		G.O. Sars	
	2007	2008	2007	2008	2007	2008	2007	2008	2007	2008	2007	2008	2007	2008	2007	2008
Mean	1.92	0.97	0.90	0.96	1.14	1.17	1.15	1.21	1.10	1.14	1.09	1.16	0.24	0.23	0.33	0.33
Median	1.94	0.97	0.91	0.95	1.15	1.15	1.17	1.19	1.11	1.12	1.11	1.14	0.24	0.23	0.34	0.34
SD	0.26	0.16	0.08	0.10	0.17	0.22	0.12	0.13	0.17	0.22	0.12	0.14	0.08	0.05	0.05	0.05
SE	0.03	0.02	0.01	0.01	0.02	0.03	0.01	0.02	0.02	0.03	0.01	0.02	0.01	0.01	0.00	0.01
CL (95%)	0.056	0.050	0.017	0.025	0.035	0.070	0.023	0.033	0.036	0.070	0.024	0.034	0.017	0.017	0.009	0.012
Minimum	1.23	0.68	0.69	0.74	0.73	0.79	0.87	0.92	0.67	0.75	0.80	0.85	0.06	0.14	0.14	0.20
Maximum	2.54	1.29	1.12	1.18	1.54	1.64	1.45	1.50	1.51	1.61	1.41	1.47	0.61	0.35	0.45	0.39
Kurtosis	-0.28	-0.86	-0.07	-0.56	-0.30	-0.72	-0.04	-0.63	-0.40	-0.73	-0.08	-0.64	6.65	-0.86	3.37	0.85
Skewness	-0.17	0.20	-0.31	0.21	-0.10	0.34	-0.19	0.22	-0.12	0.33	-0.10	0.32	1.74	0.22	-1.02	-1.21
n	87	40	101	67	87	40	101	67	87	40	101	67	87	40	101	67

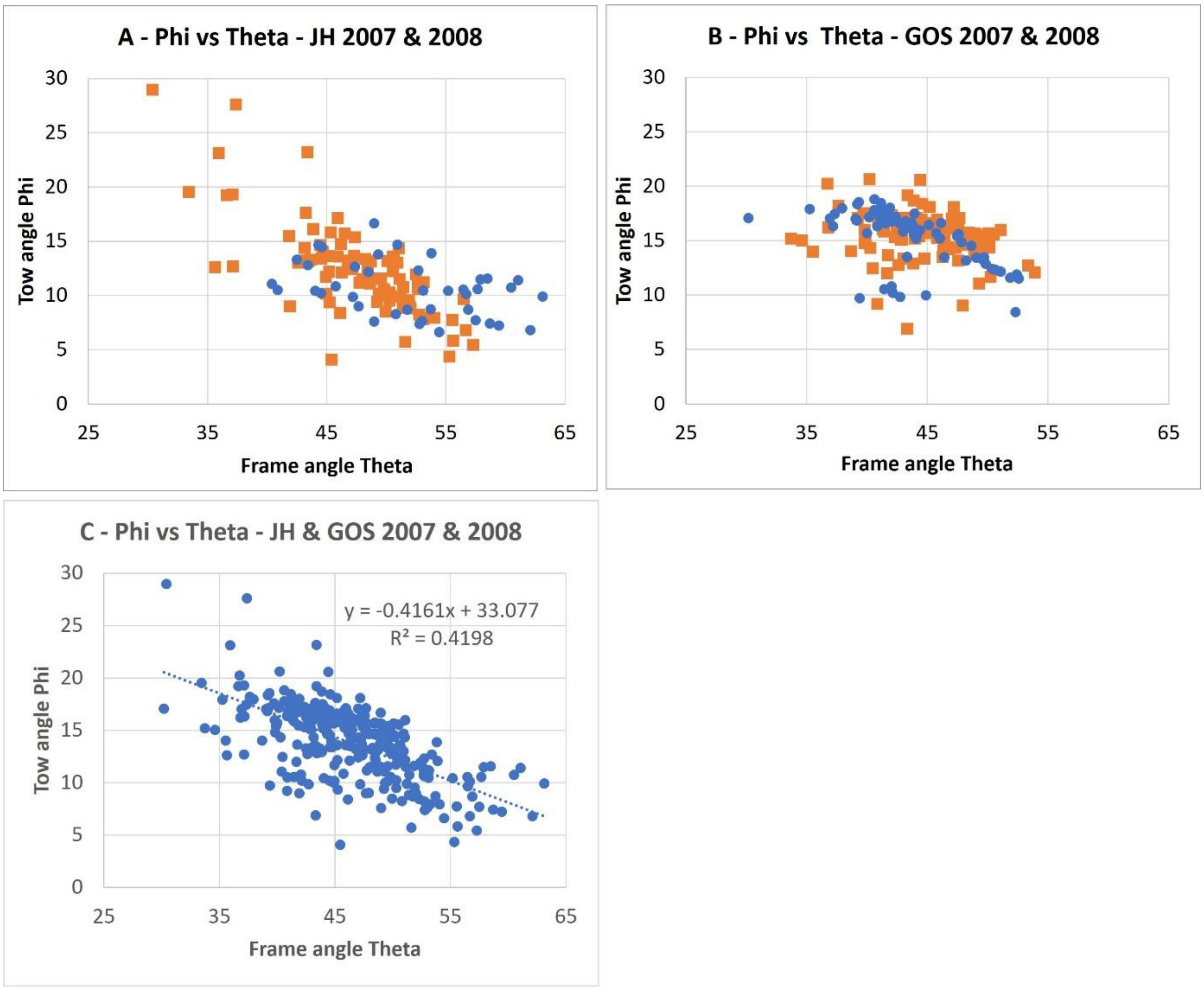

Fig. S-10. Relationship between tow angle φ and frame angle θ for individual MOCNESS nets in the data sets for RVs Johan Hjort (A) and G.O. Sars (B) in 2007 (square symbols) and 2008 (circular symbols). (C) Data for both ships and years combined.

The MOCNESS was towed with similar speed from the two vessels, reflected by mean flow count and mean net speed (Table S-8). The recorded flow count for Johan Hjort in 2007 was twice as high as in 2008, reflecting the double counts; with the 2007 values divided by 2, the mean values were near identical in the two years (0.96 and 0.97 per 4 seconds). The net speed varied by a factor ~2 in each of the data sets for the two ships, with an overall range from 0.73 to 1.64 m s^{-1}. This corresponds to 1.4 – 3.2 knots, which is well above the targeted speed of 1 – 2 knots. The mean net speeds (1.14 – 1.21 m s^{-1}) correspond to 2.2 – 2.4 knots. Net speed was strictly correlated to flow count (R^2 = 0.95 – 1.00), as was horizontal speed to net speed (R^2 = 0.99). This demonstrates that the correction for off-axis orientation of the flowmeter (see Fig. S-8) had little effect on the variability of the results derived for net speed. Horizontal speed is the cosine function of φ multiplied by net speed, which for low values of φ is nearly 1.0 (0.98-0.97 for φ values of 10-15).

The vertical speed is the sine function of net speed (which is the basis for calculating φ, see Fig. S-8) and was significantly higher for GOS than JH (0.33 versus 0.23-0.24 m s^{-1}). This difference reflected the higher values of φ for GOS (Table S-8) and the higher vertical towing rate (Fig. S-9, interpreted to reflect higher winch speed).

The frame angle θ was positively correlated with towing speed reflected by the flow counts from the flowmeter (per 4-seconds; Fig. S-11). This is as expected: with higher towing speed, the MOCNESS frame is oriented flatter (more horizontally) in the water. The flow count is directly related to the net speed (NV) through water, by multiplying with the flow factor (m per count). The data from both ships showed strict relationships, although the data points and regression line were positioned higher (higher θ) for the data from 2008 than 2007 and tended also to be higher for Johan Hjort than for G.O. Sars (Fig. S-11). The reason for the difference between the ships could be the higher vertical speed for the MOCNESS operated by G.O. Sars, giving the towed frame an upward momentum, which would lower θ.

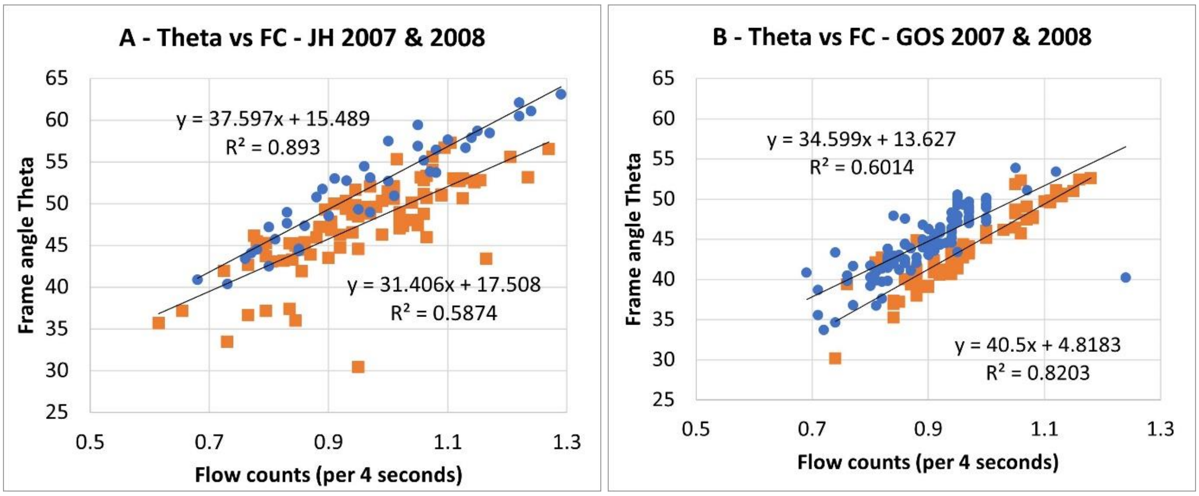

Fig. S-11. Relationships between frame the angle θ (see Fig. S-8) and relative towing speed (flow counts per 4-seconds) of MOCNESS operated during cruises with Johan Hjort (A) and G.O. Sars (B) in 2007 (square symbols) and 2008 (circular symbols). The flow counts for Johan Hjort in 2007 have been divided by 2 due to double counts.

Volume filtered by a MOCNESS net is the product of net speed (NV) and effective mouth area (MA) of the net opening. MA is the projection of the frame onto the plane perpendicular to the towing direction and is proportional to the cosine function of the sum of the two angles θ and φ (Fig. S-8A). The increase in θ with increased towing speed leads to a decrease in the projected MA through the cosine function. The decrease in MA with increasing towing speed is pronounced and counters the effect of increased NV for the calculated volume filtered per unit time (Fig. S-12). Thus, MA decreased by roughly a factor 2 for a doubling in NV, which led to stable volume filtered per time. However, the data for Johan Hjort in 2007, where the decline in MA with net velocity was less pronounced, showed an increasing trend in volume filtered per time (Fig. S-12A).

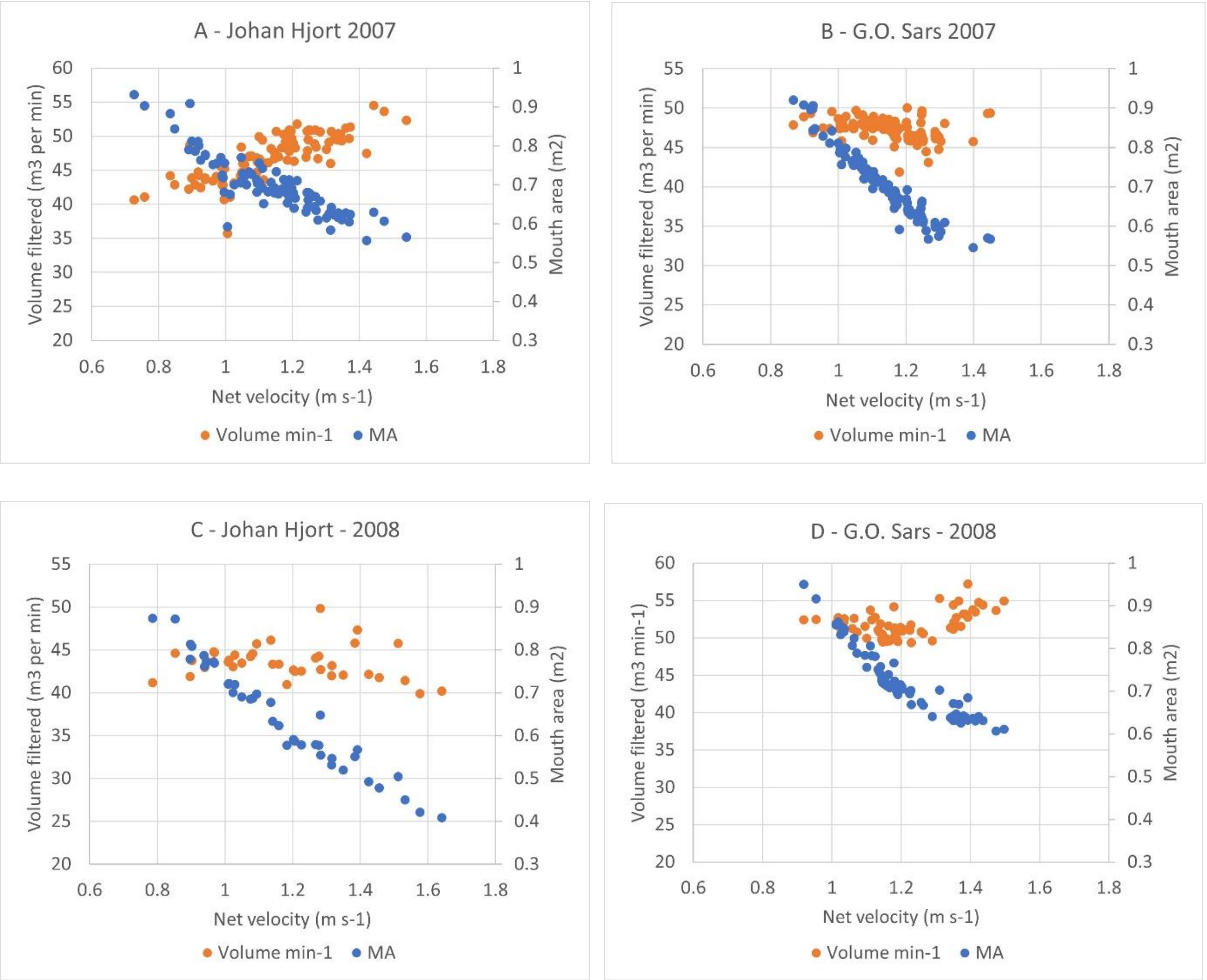

Fig. S-12. Relationships between mouth area (MA) of the effective net opening (y-axis on the right) and calculated volume filtered per time (y-axis on the left) versus net velocity for MOCNESS profiles from cruises with Johan Hjort in 2007 (A) and 2008 (C) and G.O. Sars in 2007 (B) and 2008 (D).

The calculated volume filtered per time was significantly higher for G.O. Sars than for Johan Hjort in 2008 (mean values of 51.8 versus 43.6 m^3 min^{-1}, $p < 0.001$; Table S-8) but not in 2007. This reflected lower θ and lower sum of θ and φ (despite higher φ) for G.O. Sars in 2008, which gave higher cos (θ + φ) and correspondingly higher MA. Despite the higher volume filtered per time, the total volume filtered by MOCNESS nets was lower for G.O. Sars, reflecting the higher vertical velocity and steeper trajectory up through the water column (higher φ).

Effect of change in flow factor on calculated volume filtered

We reprocessed the data for RV G.O. Sars by using flow factors (FF) 4.5 and 6.0 m per count. The reprocessing did not affect the angle θ and flow counts, which are measured and independent of the FF used. However, the use of different FFs effected the calculated tow angle φ and volume filtered. Using the total data sets, including net 0 (decent phase with negative angles), there was a near perfect correlation between values of φ calculated with the

two FFs (Fig. S-13). The values of φ were lower for the higher FF, with mean values of 15.4 vs 11.7 for FFs 6.0 and 4.5, respectively.

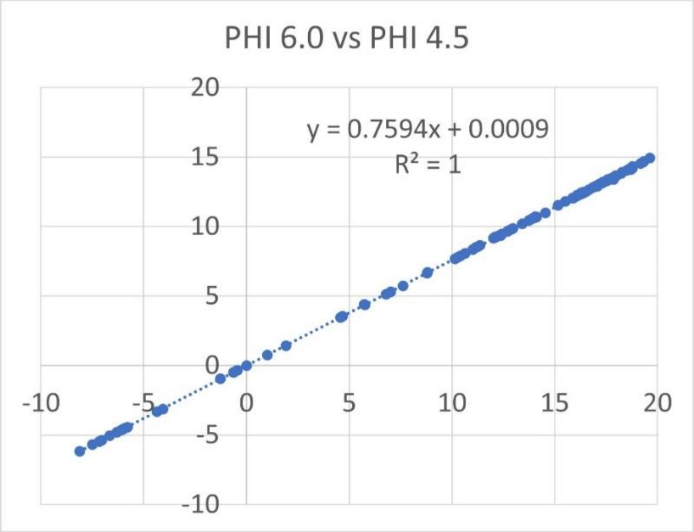

Fig. S-13. Relationship between tow angle φ calculated for the MOCNESS data from G.O. Sars in 2008 using flow factors of 4.5 (x-axis) and 6.0 (y-axis).

The reason for the lower φ for higher FF lies with geometry and Pythogoras, as illustrated in Fig. S-14. Vertical speed (VV) is calculated from measured pressure and is independent of FF. Net speed, on the other hand, is directly affected by FF. Thus, higher FF gives higher NV (a longer hypotenuse) that 'meets' the constant vector for VV at a lower angle φ. We note that φ is calculated as a sine function from NV and VV (VV = NV sin(φ)).

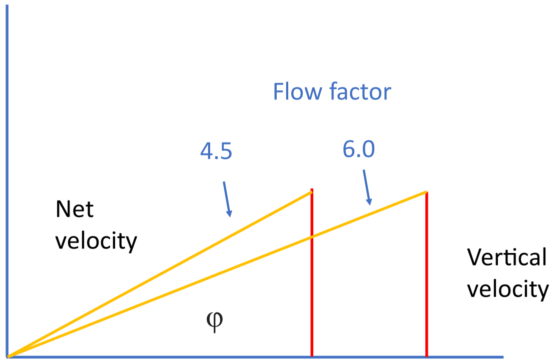

Fig. S-14. Geometric configuration illustrating the effect of flow factor on estimated tow angle φ. With higher flow factor (6.0 vs 4.5), the vector for net speed meets the vertical velocity component (which is measured and not dependent on flow factor) further out at a lower angle to the horizontal.

The two sets of estimated volume filtered by individual nets using FF 4.5 and 6.0 (m per count) for the G.O. Sars cruise in 2008 were strictly linearly related with a near perfect correlation (Fig. S-15). However, the regression slope (1.47) was higher than the ratio 1.33 between the FF values (6.0/4.5). Thus, while there is strong linearity in calculated volume filtered with changing FF, there is not strict proportionality from the ratio of FFs.

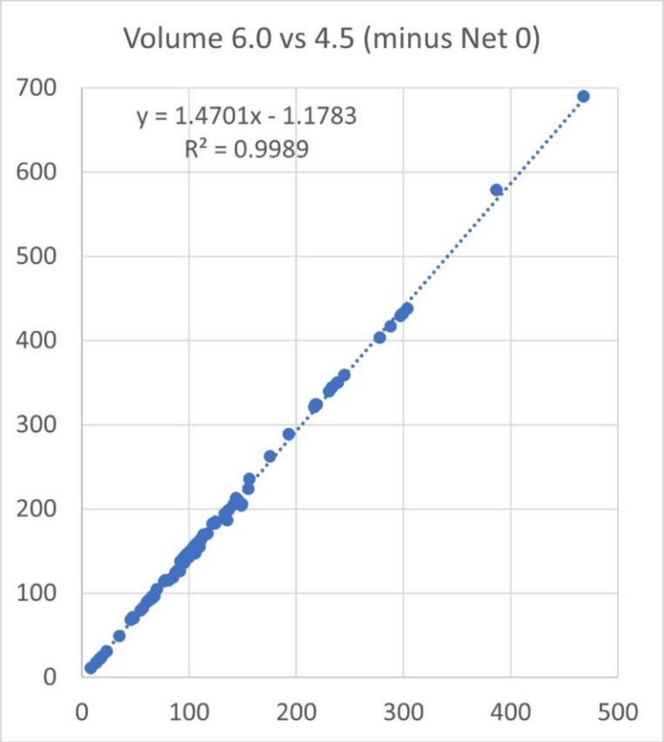

Fig. S-15. Relationship between volume filtered by MOCNESS nets (excluding net 0) calculated with FF 6.0 (y-axis) and 4.5 (x-axis) for data from cruise with G.O. Sars in 2008.

The reason for this extra effect on volume filtered lies in the effect of FF on the tow angle φ, with higher FF giving lower φ (Fig. S-14). The mouth area (MA) of the net is a cosine function of the sum of θ and φ. For lower φ with high FF, the projected mouth opening becomes larger (cos(θ+φ) is higher for lower φ with constant θ). With the numbers for the G.O. Sars 2008 cruise (θ = 43.7, φ = 15.37 and 11.67 for FF 4.5 and 6.0, respectively), the effect on the cosine function is from value 0.514 to 0.568 for FF 4.5 and 6.0, respectively. The ratio for the increased mouth area is 1.106, which is nearly identical to the ratio for the higher volume above the proportionality factor of 1.33 (1.47/1.333 = 1.103).

The data for Johan Hjort in 2007 was recalculated with FF 2.25 due to the issue of double counts. Compared to the original data where FF 4.5 was used, the new volumes were strongly correlated (R^2 = 0.99) but with an average ratio of 2.31 between the old (FF 4.5) and new (FF 2.25) volumes. Again, the reason for the higher ratio than the proportionality factor 2 lies with Pythagoras (Fig. S-14). Using the mean values (θ, net and vertical speed) for Johan Hjort in 2007 with FF 2.25 (Table S-8), and twice the net velocity in the case of FF 4.5, the difference in calculated φ (12.1 and 6.0) gives a difference of 1.17 as ratio for the mouth area (0.70 and 0.83 for FF 2.25 and 4.5, respectively). This additional effect of higher mouth area due to

lower φ comes in addition to the proportionality factor 2 for the FF ratio. However, factor 2 is not quite correct since the effect of φ on off-axis flowmeter correction (see Fig. S-8B) has been ignored. Taking this into account through the equation for net speed (NV = FNC x FF / R, where R is the cosine function of θ + φ - 45), the factor becomes 1.96 in this case with FFs 2.25 and 4.5. Combining the two factors (1.17 x 1.96) gives a factor of 2.29 for the difference in calculated volume going from FF 2.25 to 4.5, which is close to the average ratio of 2.31 for the data for the MOCNESS tows in 2007.
